# Ultrasound (US)-activated redox dyshomeostasis therapy reinforced by immunogenic cell death (ICD) through a mitochondrial targeting liposomal nanosystem

**DOI:** 10.7150/thno.62984

**Published:** 2021-09-13

**Authors:** Junjie Ren, Jing Zhou, Han Liu, Xiaodan Jiao, Yang Cao, Zhigang Xu, Yuejun Kang, Peng Xue

**Affiliations:** 1 State Key Laboratory of Silkworm Genome Biology, School of Materials and Energy, Southwest University, Chongqing 400715, China.; 2 Chongqing Key Laboratory of Ultrasound Molecular Imaging, Institute of Ultrasound Imaging, Second Affiliated Hospital, Chongqing Medical University, Chongqing 400010, China.

**Keywords:** drug delivery, liposome, targeting, redox dyshomeostasis, sonodynamic therapy

## Abstract

**Introduction:** An imbalance in redox homeostasis consistently inhibits tumor cell proliferation and further causes tumor regression. Thus, synchronous glutaminolysis inhibition and intracellular reactive oxygen (ROS) accumulation cause severe redox dyshomeostasis, which may potentially become a new therapeutic strategy to effectively combat cancer.

**Methods:** Mitochondrial-targeting liposomal nanoparticles (abbreviated MLipRIR NPs) are synthesized by the encapsulation of R162 (inhibitor of glutamate dehydrogenase 1 [GDH1]) and IR780 (a hydrophobic sonosensitizer) within the lipid bilayer, which are exploited for ultrasound (US)-activated tumor dyshomeostasis therapy reinforced by immunogenic cell death (ICD).

**Results:** R162 released from MLipRIR NPs disrupts the glutaminolysis pathway in mitochondria, resulting in downregulated enzymatic activity of glutathione peroxidase (GPx). In addition, loaded IR780 can generate high levels of ROS under US irradiation, which not only interrupts mitochondrial respiration to induce apoptosis but also consumes local glutathione (GSH). GSH depletion accompanied by GPx deactivation causes severe ferroptosis of tumor cells through the accumulation of lipid peroxides. Such intracellular redox dyshomeostasis effectively triggers immunogenic cell death (ICD), which can activate antitumor immunity for the suppression of both primary and distant tumors with the aid of immune checkpoint blockade.

**Conclusions:** Taking advantage of multimodal imaging for therapy guidance, this nanoplatform may potentiate systemic tumor eradication with high certainty. Taken together, this state-of-the-art paradigm may provide useful insights for cancer management by disrupting redox homeostasis.

## Introduction

Redox homeostasis and signaling, as innate defense mechanisms, are regarded as essential components for the maintenance of the physiological steady state of cells 1, 2. Disturbances in intracellular redox always have a major effect on cell functions because the oxidative stress response system and relevant signaling pathways are extremely sensitive to the redox environment 3. In general, redox homeostasis is attained by the strict regulation of both reactive oxygen species (ROS) production and scavenging in living cells 4. Specifically, ROS are highly reactive oxygen-derived molecules that cause damage to nucleic acids, proteins and lipids for cell signaling, biosynthetic processes and host defense. Conversely, excessive ROS activate the antioxidant defense (AOD) system and are further consumed through various metabolic reactions occurring in mitochondria, peroxisomes and the endoplasmic reticulum 5, 6. For instance, compared with healthy normal cells, slightly elevated ROS in tumor cells play an important role in cancer angiogenesis, metastasis and survival by causing deoxyribonucleic acid (DNA) damage and inducing genome inconsistencies 7, 8. In this regard, the establishment of an intracellular redox imbalance via ROS accumulation and AOD deactivation is expected to suppress tumor development, and this thriving strategy is believed to be clinically relevant for future drug development 9.

Elevated glutaminolysis in mitochondria contributes to redox homeostasis in tumor cells, and the biosynthesis and energetics provided by this signaling pathway are critical for the support of tumor growth 10, 11. Specifically, glutamate, initially derived from glutaminase (GLS)-mediated deamination of glutamine, can be converted to α-ketoglutarate (α-KG) by either glutamate dehydrogenase 1 (GDH1) or nonammonia-producing aminotransferases 12. As an intermediate product of the tricarboxylic acid cycle (TCA) cycle, α-KG is the source of anabolic nitrogen/carbon skeletons for the synthesis of amino acids, nucleotides and lipids 13. Previous reports have revealed that the GDH1 expression level is significantly increased in the late stage of breast or lung cancer in comparison to normal tissues, promoting the conversion of glutamate to α-KG in a typical glutaminolysis pathway 14, 15. In addition, the endogenous antioxidant enzyme glutathione peroxidase (GPx), as the first line of AOD, is important for the maintenance of intracellular redox homeostasis via the removal of intracellular hydroperoxide and protection of lipids from peroxidation 16, 17. Fumarate, the subsequent metabolite of α-KG, can directly bind to and upregulate the activity of GPx, which further initiates Nrf2 antioxidant signaling 10. Therefore, attenuating the activity of GDH1 by lentiviral short hairpin (sh) RNA or small molecule inhibitors would reduce the intracellular level of fumarate and subsequently disrupt redox homeostasis and release the inhibitory tumor growth signal 18, 19. The GDH1 inhibitor R162, as a purpurin analog, is capable of reducing intracellular fumarate levels, attenuating GPx activity and disabling the AOD mechanism to suppress tumor cell proliferation 20, 21. In addition to its tumor antiproliferative potential, R162 also exhibits good biocompatibility without causing significant toxicity toward normal tissues. However, R162 is unable to target mitochondria, where the glutaminolytic pathway takes place, and such nonspecificity dramatically weakens its performance in the suppression of GDH1 activity. In addition, the curative effect of glutaminolysis inhibition alone is not always sufficiently satisfactory, and adjunctive therapies are suggested to promote antitumor efficacy 22. Therefore, it is strongly recommended that a multifunctional therapeutic platform be developed not only to achieve the targeted delivery of R162 but also to augment redox dyshomeostasis through ROS accumulation.

Sonodynamic therapy (SDT), as an emerging and prosperous therapeutic strategy, has attracted increasing attention for tumor theranostics due to its merits of high tissue penetration capacity, minimal invasiveness, high controllability and low cost 23-26. On the one hand, ultrasound (US) exposure is devoted to clinical diagnostic imaging; on the other hand, acoustic luminescence from the US-mediated cavitation effect activates sonosensitizers to generate ROS, thus inducing cell apoptosis/necrosis caused by DNA fragmentation, cytoskeletal shrinkage and chromatin condensation 27, 28. In addition, a high level of ROS depletes the antioxidant GSH, causing lipid peroxidation and leading to the initiation of ferroptosis 29, 30. Among various types of organic and inorganic sonosensitizers, IR780, a lipophilic heptamethine dye with a peak optical absorption at 780 nm, is considerably appealing for clinical practice owing to its high fluorescence quantum yield, elevated US-triggered ROS generation efficiency, excellent aqueous stability and preferential accumulation in tumors 31-33. However, IR780 molecules themselves cannot actively target mitochondria, where the SDT efficacy can be maximized by directly increasing mitochondrial ROS levels to release pro-apoptotic factors 34, 35. Moreover, IR780-mediated fluorescence imaging for therapeutic guidance would suffer from nonspecific accumulation of IR780 molecules within tumorous tissue 36. Alternatively, the incorporation of IR780 into nanoparticles (NPs) can effectively improve its performance in tumor theranostics *via* enhanced permeation and retention (EPR)-mediated tumor targeting and versatile integration of functionality 37. In addition, a high level of cytotoxic ROS may trigger immunogenic cell death (ICD), which can effectively stimulate the immune response by releasing tumor-associated antigens (TAAs) and damage-associated molecular patterns (DAMPs), followed by the promoted recruitment of antigen presenting cells (APCs) with high antigen presentation activity to potentiate tumor immunotherapy 38-40. Furthermore, an anti-programmed cell death-ligand 1 (anti-PD-L1) antibody, as an immune checkpoint inhibitor, can be incorporated to propagate the anticancer immunity of ICD 41-43.

**Scheme 1:**
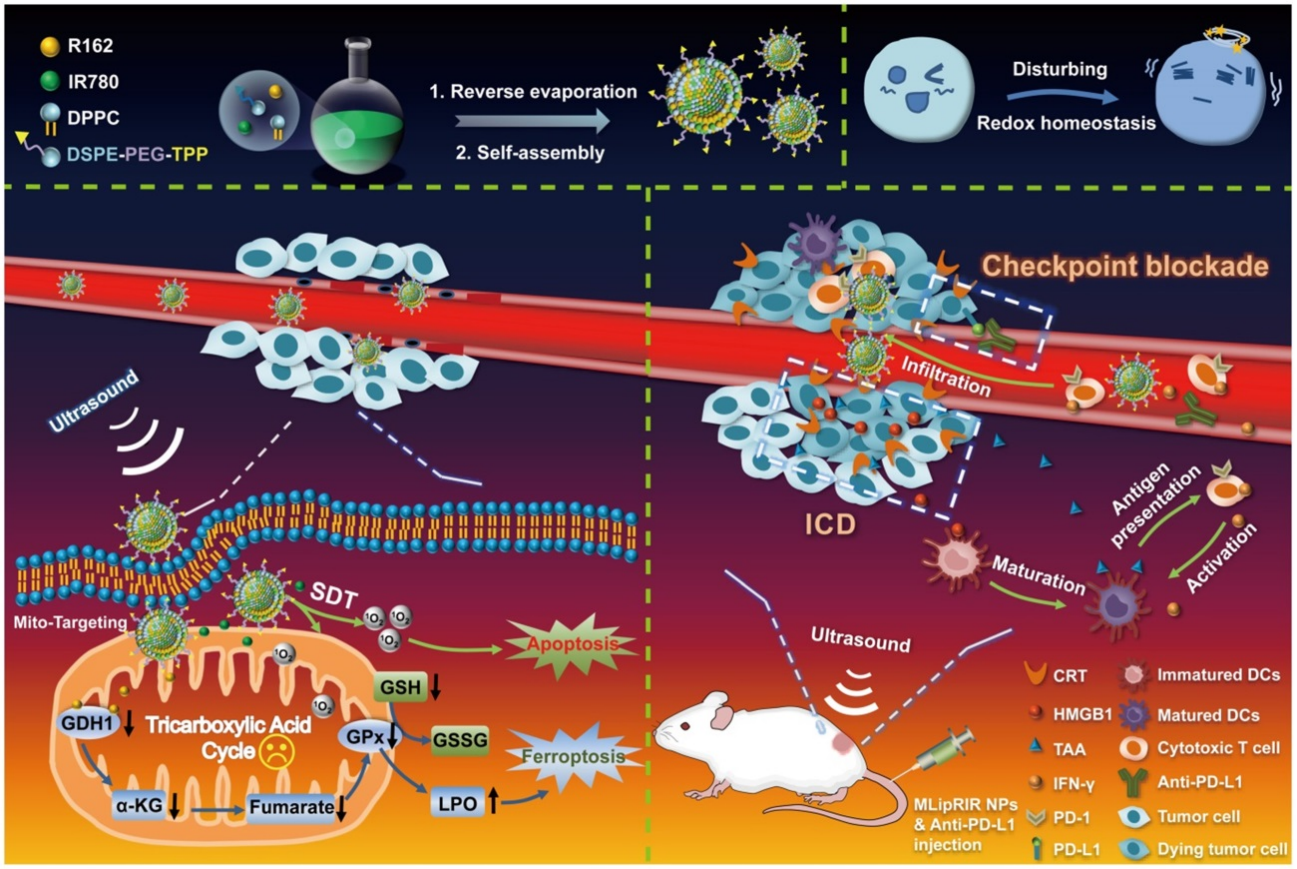
Diagram of the synthetic procedure used for MLipRIR NPs for US-activated tumor dyshomeostasis therapy. MLipRIR NPs were synthesized from the self-assembly of drug-loaded TPP-phospholipids upon reverse evaporation. After intravenous administration, MLipRIR NPs could rapidly accumulate in cancerous tissue by virtue of the EPR effect and be internalized into tumor cells. The mitochondrial targeting moiety of TPP further conducted MLipRIR NPs to the corresponding organelle, and the released R162 into the mitochondrial matrix targeted the disruption of glutaminolysis metabolism. Provided by US irradiation, ROS-mediated apoptosis of tumor cells was induced by IR780-mediated SDT. Moreover, GPx activity attenuated by glutaminolysis inhibition and GSH deprivation caused by ROS accumulation collectively resulted in severe ferroptosis. Importantly, redox dyshomeostasis effectively triggered ICD by the released DAMPs, which could synergize with PD-L1 checkpoint blockade to boost antitumor immunity and render complete eradication of both primary and distant tumors.

Taking into account the substantial roles of mitochondrial glutaminolysis and ROS levels in the maintenance of redox hemostasis, multifunctional NPs were, for the first time, successfully developed by coencapsulation of R162 and IR780 molecules into mitochondria-targeting liposomes to form Mito@Lip/R162/IR780 (abbreviated MLipRIR NPs) (**Scheme 1**). Triphenylphosphonium (TPP), a mitochondrial targeting moiety, was conjugated onto the phospholipid for the self-assembly of liposomes. Upon passive enrichment of MLipRIR NPs at the tumor site, the nanodrug can be rapidly internalized into the cytoplasm and further accumulate in the mitochondrial region 44. Next, R162 released from the nanocarrier diffused into the mitochondrial matrix and disrupted glutaminolysis metabolism to disable the AOD mechanism. Conversely, US-triggered ROS generation mediated by IR780 promoted oxidative stress and caused irreversible cell apoptosis. The resultant GSH deprivation combined with GPx activity attenuation further induced ferroptosis and aggravated redox imbalance. Moreover, cytotoxic ROS and intracellular redox dyshomeostasis could effectively trigger ICD, which synergized with immune checkpoint blockade to boost antitumor immunity *in vivo*. In addition, fluorescence/photoacoustic (FL/PA) imaging with excellent temporospatial resolution provided important evidence of maximum nanodrug enrichment in tumorous tissue, which facilitated optimization of the time point for US irradiation. Collectively, this state-of-the-art paradigm underscores the clinical potential of disturbing redox homeostasis mediated by mitochondrial-targeting liposomal NPs by virtue of deactivating the glutaminolytic pathway and elevating intracellular ROS levels.

## Experimental Section

### Materials

The 1,2-dipalmitoyl-sn-glycero-3-phosphatidylcholine (DPPC, >99%), 1-(3-dimethylaminopropyl)-3-ethylcarbodiimide hydrochloride (EDC, >98%), 1,2-distearoyl-sn-glycero-3-phosphoethanolamine-N-[methoxy(polyethylene glycol)-2000] (DSPE-PEG_2000_), (4-carboxybutyl)triphenylphosphonium bromide (TPP, >98%), 1,2-distearoyl-sn-glycero-3-phosphoethanolamine-N-[amino(polyethylene glycol)-2000] (DSPE-PEG_2000_-NH_2_), 1,3-diphenylisobenzofuran (DPBF, >97%) and 2-(4-morpholinyl)ethanesulfonic acid hydrate (MES) buffers were purchased from Shanghai Aladdin BioChem Technology Co., Ltd. The IR-780 iodide (>95%) was supplied by Adamas Reagent Co., Ltd. (China). The N-hydroxysuccinimide (NHS, >98%) was acquired from Shanghai Macklin Biochemical Co., Ltd. (China). Trichloromethane was obtained from Chongqing Chuandong Chemical Co., Ltd. (China). Ferrostatin-1 was provided by Shanghai Yuanye Bio-Technology Co., Ltd. (China). The 3-(4,5-dimethylthiazol-2-yl)-2,5-diphenyltetrazolium bromide (MTT), Triton^TM^ X-100, 2,2,6,6-tetramethyl-4-piperidone hydrochloride (TEMP) and dimethyl sulfoxide (DMSO) were from Millipore Sigma (USA). The BCA protein, JC-1 mitochondrial membrane potential (MMP), ATP assay kit, glutathione peroxidase (GPx) activity, glutathione, lipid peroxidation (MDA), and reactive oxygen species (ROS) assay kits and 4,6-diamidino-2-phenylindole (DAPI) were obtained from Beyotime Biotech Inc. (China). MitoTracker Green, formulated Dulbecco's modified Eagle's medium (DMEM), TrypLE^TM^ Express Enzyme, phosphate-buffered saline (PBS), fetal bovine serum (FBS), the LIVE/DEAD^®^ viability/cytotoxicity kit and the Annexin V-FITC/PI apoptosis detection kit were from Thermo Fisher Scientific (USA). LiperFluo was purchased from Dojindo Molecular Technologies, Inc. (Japan). Calreticulin (CRT) and high mobility group box 1 (HMGB1) antibodies, FITC-labeled secondary antibody, mouse interferon-γ (IFN-γ), tumor necrosis factor-α (TNF-α) and the interleukin-6 (IL-6) enzyme-linked immunosorbent assay (ELISA) kit were obtained from Proteintech Group, Inc. (USA). The anti-mouse PD-L1 antibody was purchased from BioXcell (USA). The alpha-ketoglutarate (α-KG) and fumarate assay kits were from BioVision, Inc. (USA).

### Synthesis of mitochondrial-targeting phospholipid

Mitochondrial-targeting phospholipid polymer was synthesized according to a previous report 45. Briefly, PPh_3_Br-(CH_2_)_4_-COOH (TPP) (7.956 mg) was dissolved in 0.01 M MES buffer (4 mL, pH 5.4). EDC (10.45 mg) and NHS (12.42 mg) were added to the previous solution, and the mixture was stirred at room temperature for 10 min. Next, 4 mL PBS (pH = 7.2) containing DSPE-PEG_2000_-NH_2_ (5 mg) was added to the above mixture, followed by stirring at room temperature for 24 h. To remove unconjugated small molecules, the reaction solution was loaded into a dialysis bag (MWCO = 2 kDa) and dialyzed against deionized (DI) water for 48 h. Finally, mitochondrial-targeting DSPE-PEG_2000_-TPP phospholipid was lyophilized for 24 h in a freeze dryer and stored at -20 °C prior to use.

### Synthesis of MLipRIR NPs

MLipRIR NPs were synthesized via a typical reverse evaporation method 46. Specifically, DPPC (10 mg), DSPE-PEG_2000_-TPP (4 mg), cholesterol (3 mg), IR780 (3 mg) and R162 (0.54 mg) were thoroughly dissolved in trichloromethane solution (10 mL). Next, the above solution was transferred to a round-bottom flask, followed by evaporation on a rotary evaporator (100 rpm, 100 mbar) at 50 °C for 30 min. Upon complete removal of the solvent, a lipid thin film was formed on the flask bottom. Subsequently, 10 mL of PBS solution (pH = 7.4) was introduced into the previous flask containing the phospholipid thin film for hydration, and emulsification was carried out by high-intensity ultrasonication at an output power of 100 W (pulse duration = 4 s, resting interval = 5 s) for 10 min. The as-developed MLipRIR NPs were finally harvested by centrifugation (6000 rpm, 15 min), redispersion and subsequent extrusion by mini-extruders with a 200-nm membrane. In addition, LipRIR NPs without mitochondrial-targeting capacity were prepared from a mixture of DPPC (10 mg), DSPE-PEG_2000_ (4 mg), cholesterol (3 mg), IR780 (3 mg) and R162 (0.54 mg) in trichloromethane solution (10 mL), which was similarly processed by film hydration and emulsion *via* ultrasonication. All other conditions were similar to those described for the synthesis of MLipRIR NPs. The drug-loading content and encapsulation efficiency of R162 were determined using optical spectrophotometry (λ_max_ = 410 nm), and these two parameters for IR780 were simultaneously quantified based on fluorescence spectrophotometry (λ_ex_ = 650 nm, λ_em_ = 780 nm).

### Characterizations

The morphology of MLipRIR NPs was examined using a Jeol JEM-1200EXII transmission electron microscope (TEM, JEOL, Japan). Optical absorbance spectra were acquired from a UV-1800 UV/visible scanning spectrophotometer (Shimadzu Scientific Instruments, Japan). The hydrated particle size and zeta potential of the NPs were measured using a Zeta Sizer/Nano ZS90 analyzer (Malvern Panalytical, UK). US irradiation was conducted using an Intelect TranSport Ultrasound Unit (Chattanooga Group, Inc., USA).

### R162 release *in vitro*

To investigate R162 release from MLipRIR NPs, a 5-mL sample dispersion (10 mg/mL) in a dialysis bag (MWCO = 3500 Da) was submerged in 35 mL PBS (pH = 6.8 or 7.4). The setup was then placed in an incubator at a fixed temperature of 37 °C. At predetermined time points, 2 mL releasing medium was withdrawn to determine the drug release kinetics, and fresh medium with the equivalent volume was replenished for consistency. To investigate US-activated drug release, a sample-laden dialysis bag was exposed to periodic US irradiation (1.0 MHz, 1.5 W/cm^2^, 50% duty cycle; on: 1 min, off: 3 min for each cycle). Cumulative drug release was quantified by optical spectrophotometry (λ_max_ = 410 nm).

### US-activated ROS generation *in vitro*

A DPBF fluorescence probe was first used to monitor ROS generation during the sonodynamic process. Briefly, 40 µL DPBF (8 mM) was added to 2 mL PBS medium containing MLipRIR NPs at various concentrations. Then, the mixture was exposed to US irradiation (1.0 MHz, 1.5 W/cm^2^, 50% duty cycle) at 1-min intervals. Next, the absorption intensity at 417 nm was dynamically recorded using a spectrophotometer, and the amount of produced ROS was positively correlated with the absorption decrease. Furthermore, electron spin resonance (ESR) spectroscopy was conducted to determine the category of generated radical species during US irradiation. TEMP (1 mg/mL), as the trapping agent for the detection of singlet oxygen (^1^O_2_), was used for the ESR assay. Briefly, 50 μL MLipRIR NPs (50 µg/mL) was exposed to US irradiation (1.0 MHz, 1.5 W/cm^2^, 50% duty cycle) for 1 min in the presence of TEMP, followed by ESR spectroscopic analysis at room temperature. Additionally, reference groups of “DI water”, “MLipRIR” and “US” were tested as negative controls.

### Cellular uptake

Cellular uptake and internalization of MLipRIR NPs were investigated by both confocal laser scanning microscopy (CLSM, LSM800, Zeiss, Germany) and flow cytometry (NovoCyte TM 2060R, ACEA Biosciences, USA). Specifically, 4T1 cells in a 12-well plate (sending density: 5×10^4^ cells per well) were cultured at 37 °C for 8 h, followed by exposure to MLipRIR NPs (equivalent R162 concentration: 1 µg/mL) for a period of 0.5, 1, 2, 4 and 6 h. In parallel, cells without any treatment served as the blank group. For CLSM, these treated cells were washed with PBS three times and fixed with 4% paraformaldehyde for 20 min. Then, the cells were stained with DAPI (1 μg/mL) for 5 min, followed by rinsing with PBS three times. Finally, intracellular fluorescence was examined via CLSM. In another aspect, cells were trypsinized and subsequently resuspended in 400 µL PBS (containing Ca^2+^ and Mg^2+^), followed by analysis using a flow cytometer. The acquired data were analyzed by FlowJo (v10). To assess the mitochondrial-targeting capability of MLipRIR NPs, 4T1 cells in a 12-well plate (density: 5×10^4^ per well) were cultured at 37 °C for 8 h, followed by exposure to MLipRIR NPs (equivalent R162 concentration: 1 µg/mL) for 4 h. Subsequently, the cells were washed with PBS and stained with MitoTracker Green (500 nM) for 30 min for mitochondrial labeling. Finally, the colocalization of mitochondria and MLipRIR NPs was analyzed by CLSM.

### Biocompatibility

The biocompatibility of MLipRIR nanocarriers *in vitro* was measured using human umbilical vein endothelial cells (HUVECs) in compliance with a typical MTT cell proliferation assay. Specifically, cells in a 96-well plate (sending density: 1×10^4^ cells per well) were cultured at 37 °C for 8 h, followed by exposure to MLipRIR NPs at different concentrations (0-40 µg/mL) for 12 h or 24 h. Untreated cells served as the negative reference. After rinsing with PBS several times, 100 µL MTT solution (0.5 mg/mL) was added to each well. After 4 h of incubation, the previous MTT solution was replaced with DMSO (100 µL) for homogenization. After 15 min of gentle shaking, the optical absorption intensity (λ: 570 and 630 nm) was measured with a microplate reader (Spark 10M, Tecan, Switzerland). Finally, cell viability was calculated according to a recommended formula from the manufacturer.

### *In vitro* cytotoxicity

The Chattanooga Intelect Mobile Ultrasound Transducer (Chattanooga Co., USA) was used as the ultrasonic source for all tests both *in vitro* and *in vivo*. The US irradiation intensity of 1.5 W/cm^2^ was manually customized on the control panel. To apply the US irradiation, the transducer was placed against the bottom of a 96-well plate, which was mediated by coupling agents to maximize the acoustic interface contact. The cytotoxicity of MLipRIR NPs toward 4T1 cells was evaluated *via* a standard MTT assay *in vitro*. Briefly, 4T1 cells in a 96-well plate (seeding density: 1×10^4^ cells per well) were cultured at 37 °C for 8 h and then exposed to MLipR, MLipIR, LipRIR or MLipRIR NPs (equivalent R162 concentration: 1 µg/mL) in DMEM. After treatment for 4 h, the cells were exposed to US irradiation (1.0 MHz, 1.5 W/cm^2^, 50% duty cycle) for 1 min in the experimental groups. After incubation for another 8 h, MTT assay was conducted to determine cell viability according to the abovementioned protocol. To understand the ferroptosis induced by MLipRIR NPs, the nanodrug was administered with the addition of ferrostatin-1 (a ferroptosis inhibitor, 0.5 µg/mL), followed by the MTT assay. For lipid hydroperoxide detection, cells were prestained with 10 μM LiperFluo for 30 min, followed by various treatments and subsequent observation through CLSM. The cell apoptosis level was determined using flow cytometry. After various treatments, the cells were trypsinized, centrifuged and redispersed in PBS, followed by staining with Annexin V-FITC (5 μL) and PI (5 μL) for 15 min in the dark prior to flow cytometry.

### Live/dead cell staining assay

The cytotoxicity caused by MLipRIR NPs was further validated using a LIVE/DEAD^®^ viability/cytotoxicity assay. Briefly, 4T1 cells in a 96-well plate (seeding density: 10^4^ cells per well) were cultured at 37 °C for 8 h. Afterward, adherent cells were treated with MLipR, MLipIR, LipRIR or MLipRIR NPs (equivalent R162 concentration: 1 µg/mL) in DMEM. After treatment for 4 h, the cells were exposed to US irradiation (1.0 MHz, 1.5 W/cm^2^, 50% duty cycle) for 1 min in the experimental groups. After incubation for another 8 h, the cells were costained with calcein AM and PI in accordance with a recommended protocol, and intracellular fluorescence was examined under a fluorescence microscope (IX73, Olympus, Japan). Mitochondrial damage was monitored using membrane-permeant JC-1 dye. After various treatments, 4T1 cells were stained with JC-1 fluorescence probe (10 µg/mL) for 10 min, and intracellular fluorescence was examined using FITC (λ_em_ = 525 nm) and Cy3 (λ_em_ = 580 nm) channels with CLSM.

### ROS generation at the cellular level

A DCFH-DA probe (intracellular ROS assay) was used to evaluate US-activated ROS generation *in vitro*. Briefly, 4T1 cells in a 12-well plate (seeding density: 1×10^5^ cells per well) were first incubated at 37 °C for 8 h and then treated with MLipR, MLipIR, LipRIR or MLipRIR NPs (equivalent R162 concentration: 1 µg/mL) in DMEM. After 4 h incubation, US irradiation (1.0 MHz, 1.5 W/cm^2^, 50% duty cycle) was performed for 1 min in the experimental groups. After staining with the DCFH-DA probe (10 μM) for 30 min, the treated cells were washed with PBS and observed using CLSM.

### Glutaminolysis pathway blockade by MLipRIR NPs

To measure the intracellular α-KG level. 4T1 cells in a 6-well plate (seeding density: 5×10^5^ cells per well) were cultured at 37 °C for 8 h, followed by administration of MLipR, MLipIR, LipRIR or MLipRIR NPs (equivalent R162 concentration: 1 µg/mL) in DMEM for 4 h. US irradiation (1.0 MHz, 1.5 W/cm^2^, 50% duty cycle) was performed for 1 min in the experimental groups. After incubation for another 8 h, cell counts were determined using a hemocytometer. After lysis with cell lysis buffer, the α-KG level in each group was determined using an α-KG colorimetric/fluorometric assay kit. To measure intracellular fumarate levels, 4T1 cells received similar treatments as mentioned above, and intracellular fumarate levels in each group were detected using a fumarate colorimetric assay kit. To determine GPx activity, 4T1 cells received similar treatments as mentioned above, and GPx activity in each group was determined using a GPx activity colorimetric assay kit.

### Intracellular ATP measurement

The 4T1 cells received similar treatments as stated in the characterization of glutaminolysis pathway blockade. Afterward, the treated cells were centrifugally collected and lysed in ice-cold ATP detection sample buffer. After centrifugation at 13,000 rpm at 4 °C for 10 min, the resultant supernatant was harvested to measure intracellular ATP content using a luminescent ATP detection assay kit.

### ICD biomarker detection

The 4T1 cells received similar treatments as stated in the characterization of glutaminolysis pathway blockade. Next, the cells were fixed with paraformaldehyde (4%) for 10 min, permeated with Triton X-100 (0.2%) for 5 min and blocked with BSA (1%) at room temperature for 1 h. The permeabilization step was excluded with regard to the immunofluorescence staining of CRT. Afterward, the fixed cells were incubated with primary antibodies against HMGB1 (1.2 µg/mL) and CRT (1.9 µg/mL) at 4 °C for 12 h. Subsequently, the cells were treated with FITC-labeled secondary antibodies at 4 °C for 3 h and stained with DAPI (1 µg/mL) for 10 min. Finally, intracellular fluorescence was detected through individual detection channels using CLSM.

### Tumor xenograft establishment

Animal experiments in this study were authorized by the Institutional Animal Care and Use Committee (IACUC) of Southwest University under permission number SYXK (Chongqing) 2017-0019 and complied with the National Guide for Care and Use of Laboratory Animals (China). Briefly, the dorsal region of female BALB/c mice (18-22 g, 8 weeks of age) was subcutaneously inoculated with 100 µL saline buffer containing 4T1 cells (1×10^7^ per mL). All mice were ready for drug administration when the tumor volume reached ~250 mm^3^.

### Fluorescence imaging

To evaluate the biodistribution of MLipRIR NPs *in vivo*, BALB/c mice bearing 4T1 tumors were administered 100 μL saline containing MLipRIR NPs (3 mg/mL) via the tail vein. NIR fluorescence images were acquired at 0, 0.5, 1, 2, 4, 6, 12 and 24 h postinjection through a multifunctional imager (Fusion FX7 Spectra, VILBER, France). The average fluorescence intensity at the tumor site was quantified using the same system. Additionally, solid tumors and major organs (heart, liver, spleen, lung, and kidney) were harvested at 12 or 24 h for *ex vivo* fluorescence imaging.

### Photoacoustic imaging

Photoacoustic images of MLipRIR NPs at different concentrations (0, 62.5, 125, 250, 500 μg/mL) were taken using a VIVO 2100 LAZR imaging system (FUJIFILM Visual Sonics, Inc., Canada). To evaluate the photoacoustic imaging properties *in vivo*, BALB/c mice bearing 4T1 tumors were intravenously (*i.v.*) administered MLipRIR saline dispersion (100 μL, 3 mg/mL), and photoacoustic images were captured by scanning the tumor region at 0, 2, 4, 8, 12 and 24 h postinjection. The photoacoustic signal intensity was quantified by analyzing the acquired images using the aforementioned imaging system.

### Pharmacokinetics *in vivo*

To investigate pharmacokinetics *in vivo*, Kunming (KM) mice were intravenously injected with 100 μL R162 or MLipRIR NPs (equivalent R162 dosage at 0.5 mg/kg, in saline). At predesigned time points (0.167, 0.5, 1, 4, 6, 12 and 24 h), 150 μL whole blood was withdrawn from the retroorbital plexus, followed by protein precipitation and centrifugation (12,000 rpm, 5 min). Finally, the R162 level in blood was quantified from a standard curve through spectrophotometry.

### Hemolytic assay

Fresh blood (500 μL) was withdrawn from the orbital venous plexus of KM mice using a blood collecting vessel. Red blood cells (RBCs) were then harvested from the whole blood by centrifugation at 3000 rpm for 5 min. After rinsing with PBS five times, the purified RBCs (0.25 mL, 4% v/v, in PBS) were mixed with MLipRIR NPs (dispersion in PBS) to a final concentration ranging from 15.625 to 500 μg/mL. All the samples were incubated at 37 °C for 6 h, and the supernatant was obtained by centrifugation at 12,000 rpm for 10 min. The absorption intensity of the supernatant (λ = 570 nm) was recorded for hemolysis rate quantification via spectrophotometry using the following equation: Hemolysis (%) = (I_∞_-I_0_) / (I-I_0_) × 100%, where I, I_0_ and I_∞_ signify the absorption intensities of RBCs treated with MLipRIR NPs, PBS and DI water, respectively.

### Routine blood tests

KM mice (4-6 weeks, 25 g) were intravenously administered MLipRIR NPs (100 µL, equivalent R162 concentration at 0.5 mg/kg, in saline). On day 1, 3, 5, 7 and 14, blood was collected and analyzed using a hematology analyzer (BC-5000Vet, Mindray, China). Primary blood indices, including white blood cells (WBCs), platelets (PLTs), RBCs, hemoglobin (HGB), hematocrit (HCT), mean corpuscular volume (MCV), lymphocytes, granulocytes and monocytes, were recorded to evaluate systemic toxicity.

### Tumor suppression effect* in vivo*

BALB/c mice bearing 4T1 tumors were allocated into seven groups (n = 5 each group): (1) saline, (2) saline + US, (3) MLipR + US, (4) MLipIR + US, (5) LipRIR NPs + US, (6) MLipRIR NPs and (7) MLipRIR NPs + US. On day 0, 2 and 6, tumor-bearing mice were intravenously injected with 100 μL sample solution in all groups. In groups (3)-(6), mice were administered various agents (equivalent R162 dosage at 0.5 mg/kg, in saline). At 12 h postinjection, US irradiation (1 MHz, 1.5 W/cm^2^, 50% duty cycle) was conducted for 5 min in the applicable groups. To apply US irradiation, medical ultrasonic coupling agent was smeared on the outer surface of the hair-shaved tumor, followed by pressing the US transducer probe against skin surface. The axial length and width of the solid tumor were measured daily using a Vernier caliper, and the tumor volume was calculated using the following formula: tumor volume = (axial length) × (axial width)^2^ × 0.5. The mouse body was weighed daily during the treatment period. Tumor growth inhibition (TGI) was determined using the formula TGI = (V_C_-V_T_)/V_C_ × 100%, where V_C_ and V_T_ signify the tumor volume in the saline group and a certain treatment group, respectively. On day 14, all the mice were euthanized to harvest major organs (heart, liver, spleen, lung and kidney) and solid tumors, which were further fixed in paraformaldehyde (4% v/v) and embedded in paraffin. The embedded tissues were sliced into thin sections at a thickness of 4 μm and subjected to histological examinations, including H&E, Ki67, CRT and HMGB1 immunohistochemical staining, as well as TUNEL and immunofluorescence staining.

### PD-L1 blockade synergized suppression of distant tumors

Then, 100 µL saline buffer containing 4T1 cells (1×10^7^ per mL) was subcutaneously injected into the right dorsal region of each mouse. After six days, similar tumor inoculation in the left dorsal region of each mouse was carried out through similar subcutaneous injection. One day later, tumor-bearing mice were randomly assigned to one of five groups (n = 5 each group): (1) saline, (2) saline + US, (3) anti-PD-L1, (4) MLipRIR NPs + US and (5) MLipRIR NPs + US + anti-PD-L1. On day 0 and 3, tumor-bearing mice were intravenously injected with 100 μL sample solution in all groups, and the drug dosage of each group was constant at an equivalent R162 concentration of 0.5 mg/kg in saline. US irradiation (1 MHz, 1.5 W/cm^2^, 50% duty cycle) was conducted against the primary tumor region 12 h postinjection in groups (2), (4) and (5). Anti-PD-L1 antibody (75 μg per mouse) was intraperitoneally (*i.p.*) administered on day 1, 4, 5 and 7 in groups (3) and (5). Tumor volume and mouse body weight were monitored daily during the treatment period. On day 14, all mice were euthanized to harvest major organs (heart, liver, spleen, lung and kidney) and solid tumors, which were further sliced into thin sections at a thickness of 4 μm. Histological analysis, including H&E, Ki67, CRT and HMGB1 immunohistochemical staining, as well as TUNEL and immunofluorescence staining, was performed for histopathological examination.

### Detection of secreted cytokines

On day 8, mouse whole blood was withdrawn from the orbital venous plexus. After resting for 30 min, supernatant containing serum was isolated from whole blood through centrifugation at 1000 rpm for 15 min. Finally, proinflammatory cytokines in serum, including TNF-α, IFN-γ and IL-6, were detected using the corresponding ELISA immunoassay kit.

### Statistical analysis

All data are displayed as the mean ± standard deviation (SD). One-way analysis of variance (ANOVA) was performed for multiple group comparisons, whereas the Student's* t* test was carried out for two group comparisons. The default thresholds for statistical significance were defined as ^*^p<0.05, ^**^p<0.01 and ^***^p<0.005.

## Results and Discussion

### Synthesis and characterization of MLipRIR NPs

**Figure 1:**
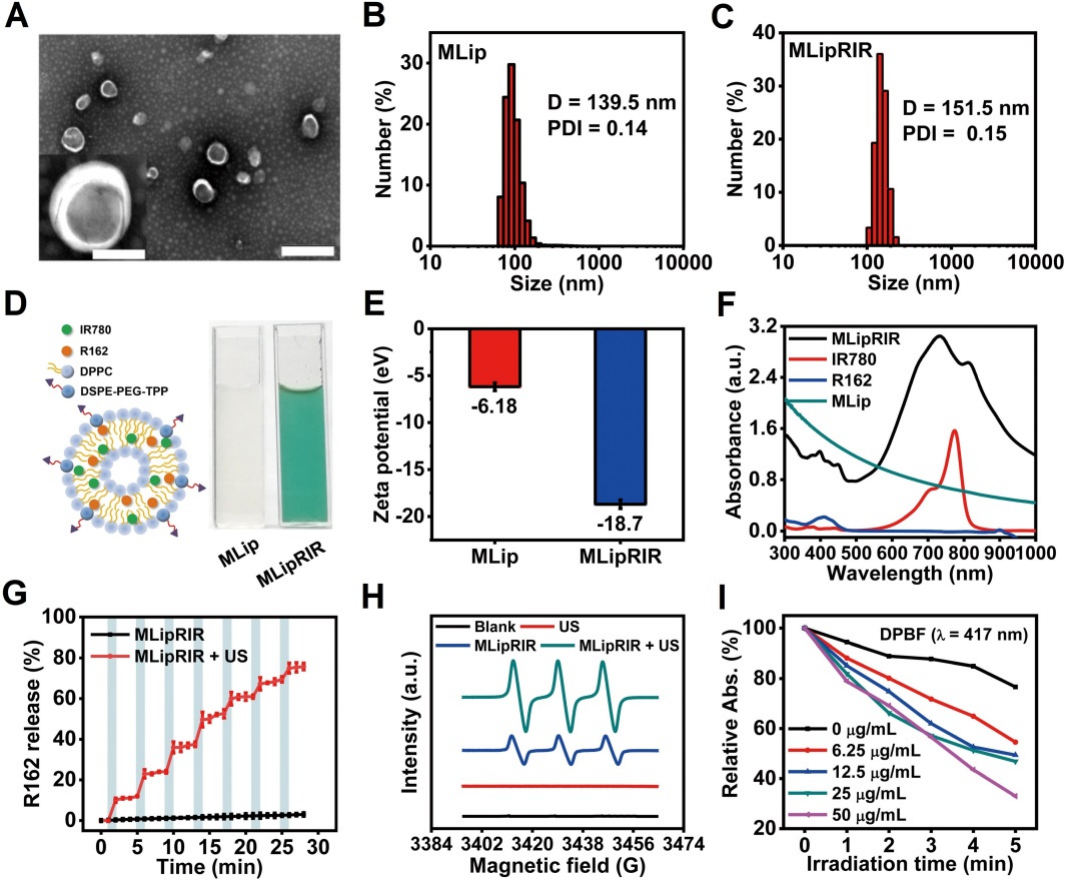
** Synthesis and characterizations of MLipRIR NPs. (A)** TEM image showing the spherical morphology of MLipRIR NPs (scale bar: 500 nm). Inset: high-magnification TEM image of single MLipRIR NPs (scale bar: 100 nm). Hydrodynamic diameter of **(B)** MLip empty nanocarrier and **(C)** MLipRIR NPs measured by dynamic light scattering (DLS). **(D)** Schematic diagram illustrating the structural composition of MLipRIR NPs, and digital photographs of MLip empty nanocarrier as well as MLipRIR NP dispersions. **(E)** Zeta potential of MLip empty nanocarriers and MLipRIR NPs. **(F)** UV-vis-NIR absorption spectra of MLip empty nanocarrier (280 μg/mL), R162 (0.9 μg/mL), IR780 (5 μg/mL) and MLipRIR NPs (300 μg/mL), indicating the successful encapsulation of R162 and IR780 into the MLip nanocarrier. **(G)** Cumulative release of R162 from MLipRIR NPs under physiological conditions (PBS, pH: 7.4, 37 ^o^C) *in vitro.* Blue hatched time slots represent the US irradiation period (1.0 MHz, 1.5 W/cm^2^, 50% duty cycle; 1 min irradiation for each cycle). **(H)** Electron spin resonance (ESR) spectra of the sample in the presence of TEMP (trapping agent of ^1^O_2_), wherein US irradiation (1 MHz, 1.5 W/cm^2^, 50% duty cycle) was carried out for 1 min. **(I)** DPBF absorption decrease in different concentrations of MLipRIR NPs under US irradiation for different periods (0-5 min).

Hydrophobic molecules of R162 (glutaminolysis inhibitor) and IR780 (sonosensitizer and fluorescence tracer) were coencapsulated into the bilayer of liposomes to form MLipRIR NPs through reverse evaporation and self-assembly. Liposomes were selected as small-molecule drug carriers since liposomal products with diversified formulations have been approved by the Food and Drug Administration (FDA) for clinical use 47. As shown by transmission electron microscopy (TEM), MLipRIR NPs with uniform distribution exhibited a representative spherical structure, and the average diameter was ~161 nm (**Figure 1A**). Moreover, a high-magnification TEM image clearly displayed the drug-encapsulated bilayer phase of liposomes, as evidenced by the bright-dark fringes of the lipid membrane. The average hydrodynamic sizes of MLip empty nanocarrier and MLipRIR NPs were 139.5 ± 5.1 nm (PDI [polydispersity index] = 0.14) and 151.5 ± 2.8 nm (PDI = 0.15), respectively, as determined by dynamic light scattering (DLS) (**Figure 1B-C**). The low PDI of MLipRIR NPs indicated a narrow size distribution, providing a large surface area, steady dynamics and good order of interaction with the physical microenvironment for biomedical applications 48. In addition, there was no significant difference in the MLipRIR size measured by TEM and DLS, suggesting excellent dispersity under aqueous conditions. Moreover, the hydrodynamic size of MLipRIR NPs was located in the effective range from 50-200 nm, which is strongly recommended to achieve the effects of EPR during on-target nanodrug delivery 49. The aqueous dispersion of MLip empty nanocarriers exhibited a translucent white color, whereas the color changed to light cyan after loading IR780 and R162 (**Figure 1D**). The long-term storage stability of MLipRIR NPs under physiological conditions was investigated by recording their hydrodynamic size change in phosphate-buffered saline (PBS), fetal bovine serum (FBS, 10%) and Dulbecco's modified Eagle's medium (DMEM, with 10% FBS) for seven days (**Figure S1**). No drastic fluctuation in the hydrated diameter was found during the observation period, implying a promising structural stability for blood circulation. The zeta potentials of MLip and MLipRIR NPs were measured to be -6.18 and -18.7 eV, respectively, and the more negatively charged surface of MLipRIR contributed to a higher NP stability and less uptake by the endothelial reticular system (RES) during circulation (**Figure 1E**) 50.

To further demonstrate successful drug encapsulation, UV-vis-NIR spectra of MLip, IR780, R162 and MLipRIR were acquired by optical spectrophotometry (**Figure 1F**). Distinct characteristic broad peaks (350-450 nm) and shoulder peaks (729/811) nm of MLipRIR NPs were assigned to R162 and IR780, respectively, providing solid evidence of effective drug loading. Compared with the shoulder peaks at 710/780 nm of free IR780, these bathochromic peaks in MLipRIR can be interpreted as the formation of IR780 dimers and oligomers, also designated as J-aggregates, which can be ascribed to the change in polarity and hydrophobic interactions inside the lipid bilayer of the liposomal structure 37. This mechanism is also applied to explain the absorption profile change of hydrophobic R162 after encapsulation into liposomal nanocarriers. The loading capacities of R162 and IR780 were determined to be 2.95% and 14.8%, respectively, on the basis of optical absorption (λ_max_ = 410 nm) and fluorescence spectrophotometry (λ_ex_/λ_em_ = 650/780 nm) (**Figure S2**). In addition, the corresponding encapsulation efficiencies of R162 and IR780 were 91.3% and 95.8%, respectively. To disrupt glutaminolysis, R162 must be sustainably released from MLipRIR and diffuse into mitochondria. Thus, the release of R162 was monitored through standard dialysis under physiological conditions (PBS, pH = 7.4, 37 °C). The cumulative release of R162 reached 18.2% and 24.4% at 24 h and 48 h, respectively. implying a slow-pace of drug release. In another aspect, US irradiation has been shown to be an effective stimulus to trigger the release of payload, relying on the formation and collapse of gas nuclei in the hydrophobic region of the lipid bilayer 51. Thus, MLipRIR NPs were exposed to “On/Off” US irradiation for seven cycles over a 28-min incubation, and the R162 release profile is depicted in **Figure 1G**. Notably, R162 release was tremendously accelerated during the course of US exposure compared with the period without any treatment. In addition, the profile of US-activated R162 release was not significantly altered during seven cycles of US irradiation, indicating a good potential for multiple administrations during practical applications. After duplicate stimulations by external US, the cumulative R162 release reached 78% after 28 min, which was remarkably higher than the 3% release from the negative control without any treatment. In addition, the cumulative release of R162 from MLipRIR NPs was further evaluated under acidic conditions mimicking the TME (PBS, pH = 6.8, 37 °C) *in vitro*. The release profile of R162 was not dramatically altered during 28 min of “On/Off” US irradiation compared with that under physiological conditions (**Figure S3**). US-activated R162 release not only enables on-demand drug administration for precise medication but also decreases adverse cytotoxicity toward healthy tissues and organs during long-term circulation.

IR780, as an efficient photosensitizer, can interact with molecular oxygen and produce ^1^O_2_ through a type II photodynamic reaction. To validate the US-activated ROS generation capacity of MLipRIR NPs, electron spin resonance (ESR) spectroscopy was employed to detect short-lived ^1^O_2_ in the presence of 2,2,6,6-tetramethylpiperidine (TEMP, trapping agent of ^1^O_2_). Compared with the blank control and US irradiation groups, weak characteristic peaks with an intensity ratio of 1:1:1 were observed in the MLipRIR NP group, implying a certain level of ^1^O_2_ generation, which could be attributed to the moderate activation of IR780 under ambient light irradiation (**Figure 1H**). In contrast, the ESR amplitude in the “MLipRIR + US” group was increased by 135%, manifesting a remarkably higher ^1^O_2_ yield under US activation. Furthermore, MLipRIR-mediated ROS generation was verified using a 1,3-diphenylisobenzofuran (DPBF) molecular probe based on ROS-induced bleaching (absorption decline at 417 nm). Overall, the absorption intensity (417 nm) of MLipRIR NPs containing DPBF decreased with a prolonged US irradiation time for up to 5 min, and the decline in the absorbance rate was positively correlated with the sample concentration from 0 to 50 µg/mL (**Figure 1I and S4**). Concentration-dependent ROS production is extremely favorable for customizing therapeutic outcomes against solid tumors.

### Cellular uptake and cytotoxicity *in vitro*

**Figure 2:**
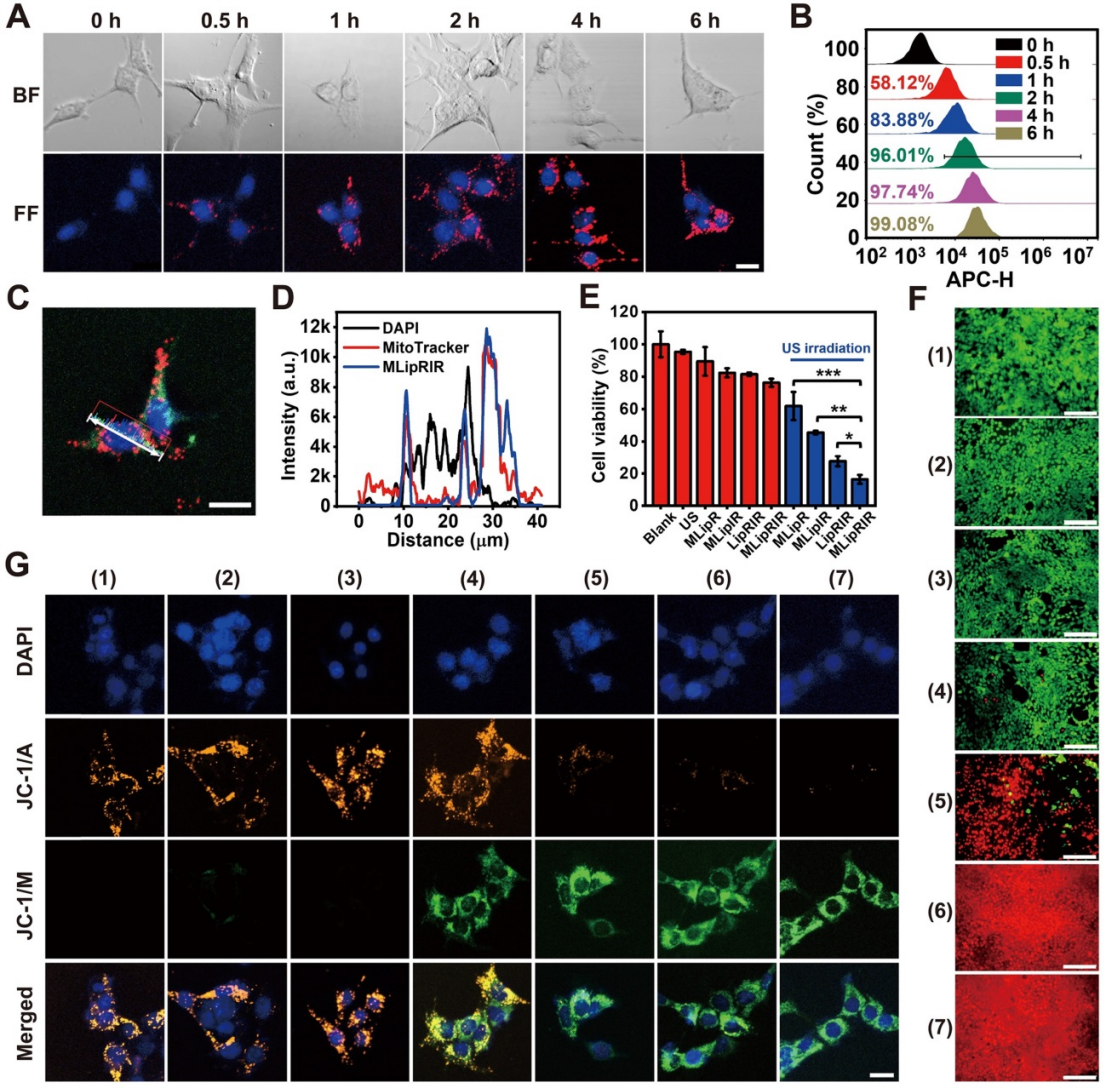
** Cellular uptake and cytotoxicity *in vitro***. **(A)** Bright-field (BF) and fluorescence-field (FF) confocal images of 4T1 cells after exposure to MLipRIR NPs (equivalent R162 concentration: 1 µg/mL) for various periods (scale bar: 20 µm). **(B)** Quantitative cellular uptake of MLipRIR NPs analyzed by flow cytometry. **(C)** Representative confocal image of 4T1 cells after incubation with MLipRIR NPs for 4 h (scale bar: 20 µm). The fluorescence of DAPI, MitoTracker Green and MLipRIR NPs is displayed by pseudocolored blue, green and red areas, respectively. **(D)** Fluorescence intensity of individual DAPI, MitoTracker Green and MLipRIR NP channels along with the white auxiliary line marked in (C). **(E)** Numerical viability of 4T1 cells after receiving various treatments (^*^p<0.05, ^**^p<0.01 and ^***^p<0.001). **(F)** Live/dead cell viability/cytotoxicity assay after 4T1 cells were treated with different regimens (scale bar: 100 µm). Live and dead cells are represented by pseudocolored green and red dots, respectively. **(G)** Fluorescence images of 4T1 cells after various treatments and staining with a JC-1 fluorescence probe (scale bar: 10 µm). The fluorescence of DAPI, JC-1 monomer (JC-1/M) and JC-1 aggregate (JC-1/A) is displayed by pseudocolored blue, green and orange areas, respectively. The sample concentration was set as an equivalent R162 dosage of 1 µg/mL. US irradiation (1.0 MHz, 1.5 W/cm^2^, 50% duty cycle) was conducted for 1 min where applicable. Groups were assigned as follows: (1) blank, (2) US, (3) MLipRIR NPs, (4) MLipR + US, (5) MLipIR + US, (6) LipRIR + US and (7) MLipRIR + US.

Rapid cellular internalization of MLipRIR NPs is the fundamental requisite for them to perform their therapeutic functions. To investigate cellular uptake, 4T1 cells (a murine mammary carcinoma cell line) were incubated with MLipRIR NPs (equivalent R162 concentration: 1 µg/mL) for different periods, and confocal microscopic images were taken under both bright-field and dark-field conditions. As shown in **Figure 2A**, intracellular enrichment of MLipRIR NPs increased with the incubation time up to 6 h, as evidenced by the progressively enhanced red fluorescence intensity. Moreover, internalized MLipRIR NPs were primarily distributed in the cytoplasm, as confirmed by the overlapping fluorescence emission area and cytoplasmic region. Flow cytometry further revealed a time-dependent endocytosis of MLipRIR NPs, which was verified by an elevated fluorescence level of IR780 over the incubation time from 0.5 h to 6 h (**Figure 2B**). In particular, the percentage of cells with internalization of a significant amount of MLipRIR NPs was 97.74% after 4 h of treatment, manifesting extremely rapid endocytic behavior (**Figure S5**). Given that R162 released from MLipRIR NPs has to diffuse into the mitochondrial matrix to inhibit glutaminolysis, high-level colocalization of internalized MLipRIR NPs with mitochondria becomes essential to obtain a maximized therapeutic outcome. Upon staining cells with MitoTracker Green, the fluorescence of MLipRIR NPs showed perfect overlap with the signals of the green-fluorescence mitochondrial tracker from the cells treated with nanoagents for 6 h, suggesting a targeted accumulation of MLipRIR NPs in the mitochondrial region (**Figure 2C-D**). In contrast, the fluorescence signal correlation between LipRIR NPs and mitochondria was extremely weak, as displayed in **Figure S6**. The admirable mitochondrial targeting property of MLipRIR NPs benefited from the presence of a TPP moiety modification in the liposomal structure. It can be speculated that the conjugation of cationic TPP may induce the “ponton sponge effect” in the acidic lysosomal matrix to cause destructive osmotic swelling, which may account for the probable escape of MLipRIR NPs from endocytosis by lysosomes and their subsequent enrichment in mitochondria. Such a lysosomal escape mechanism has also been validated in previous well-established TPP-modified liposomal systems 52, 53.

Next, the cytotoxic effect caused by MLipRIR NPs was investigated toward both human umbilical vein endothelial cells (HUVECs, a normal somatic cell line) and 4T1 cells. As shown in **Figure S7**, the viability of both HUVECs and 4T1 cells was negatively correlated with the concentration of MLipRIR NPs after administration for 12 and 24 h. It is also worth noting that MLipRIR NPs led to a more significant inhibitory effect on 4T1 cell growth when the equivalent R162 concentration exceeded the threshold of 2 µg/mL. In particular, the viability of HUVECs and 4T1 cells was 69.1% and 46.3%, respectively, after 12 h of treatment with MLipRIR NPs (equivalent R162 concentration: 8 µg/mL). Compared with normal cells, tumor cells are more sensitive to disturbances in glutaminolysis-mediated redox homeostasis, owing to their enhanced and abnormal metabolic activities 18, 54. Thus, such intrinsic selective cytotoxicity of MLipRIR NPs against tumor cells over normal cells is extremely desirable for systemic administration, with minimized adverse side effects.

Inspired by the advantageous properties of US-activated ROS generation, US-triggered R162 release and mitochondrial targeting capacity, the antitumor effect mediated by MLipRIR NPs was first evaluated via a standard MTT assay *in vitro*. The 4T1 cells were treated with various agents for 4 h, and US irradiation was conducted in the applicable groups. As shown in **Figure 2E**, moderate cytotoxicity was caused by MLipR, MLipIR or MLipRIR NPs, with cell viability over 75% in the groups without US induction. In contrast, after US irradiation (1.0 MHz, 1.5 W/cm^2^, 50% duty cycle) for 1 min, the cell viability was tremendously decreased to 61.9%, 45.5%, 27.6% and 16.4% in the MLipR, MLipIR, LipRIR and MLipRIR NP groups, respectively. The enhanced cytotoxicity in the MLipR and MLipIR NP groups might be attributed to glutaminolysis pathway dysfunction and sonodynamic effects mediated by accelerated R162 release and US-activated ^1^O_2_ production, respectively. Furthermore, LipRIR NPs contributed to a more deleterious cell killing effect, arising from enhanced redox dyshomeostasis caused by a combined SDT and glutaminolysis disturbance. The most significant tumor cell eradication was observed in the MLipRIR NP group due to the superior mitochondrial targeting properties of the MLipRIR NPs. We speculated that there are three favorable aspects for MLipRIR NPs to be enriched around mitochondria to fulfill their functions. First, R162 released from mitochondria has more opportunities to diffuse into the target organelle matrix to maximize glutaminolysis disturbance. Second, the mitochondrial area occupies a high level of local oxygen, which favors intensified US-triggered ROS generation 55. Finally, ROS produced in mitochondria consistently results in more cytotoxicity than ROS produced in other cytoplastic regions, considering the crucial role of mitochondrial ROS in molecular signal transduction and apoptotic regulation 56. A live/dead cell viability assay was further conducted to visually differentiate viable and dead cells after various treatments (**Figure 2F**). A certain level of cell destruction was clearly observed in the “MLipR + US” and “MLipIR + US” groups, as evidenced by the scattered red fluorescence dots within the viewing area. In contrast, almost exclusive red fluorescence was observed in the “LipRIR + US” and “MLipRIR + US” groups, indicating massive cell death caused by combined glutaminolysis inhibition and SDT. These fluorescence staining findings were in good accordance with the results from the MTT assay, encouraging their further validation *in vivo*.

Given that both glutaminolysis disturbance and US-triggered oxidative stress disrupt mitochondrial function, organelle health was thereby monitored by measuring the mitochondrial membrane potential (MMP), the alteration of which is closely related to the opening of the mitochondrial permeability transition pore for the release of apoptosis-associated ions or small molecules. Therefore, JC-1 dye, as a fluorescent MMP indicator, was exploited to assess the mitochondrial destruction status of cells after the different treatment regimens. In mitochondria with normal MMP polarization, JC-1 monomers (JC-1/M) form J-aggregates with a maximum fluorescence emission of 585 nm. In contrast, such aggregation would not occur in damaged mitochondria with MMP depolarization, where JC-1 dye is present as a monomer with a maximum fluorescence emission of 515 nm. As shown in **Figure 2G**, there was no obvious mitochondrial damage after applying US irradiation or MLipRIR alone. In comparison, mitochondrial health exhibited different degrees of deterioration in the other experimental groups, as verified by the increase in JC-1/M and decline in JC-1 aggregates (JC-1/A). Distinctly, almost complete disappearance of JC-1/A fluorescence along with the strongest JC-1/M fluorescence was observed in the tumor cells treated with “MLipRIR + US”, suggesting the most remarkable mitochondrial damage caused by organelle-targeted SDT and glutaminolysis inhibition. MMP depolarization usually causes the release of cytochrome c, which serves as the prominent causal factor in the early stage of apoptosis. In this regard, MLipRIR-mediated tumor cell damage was evaluated using an Annexin V-FITC/PI apoptosis assay kit (**Figure S8**). Distinct from the other control groups, as expected, the highest apoptotic cell percentage of 27.04% was attained in the “MLipRIR + US” group. Nevertheless, we speculated that unrevealed routes may have contributed to cell death in addition to the apoptosis pathway.

### MLipRIR-mediated glutaminolysis inhibition

**Figure 3:**
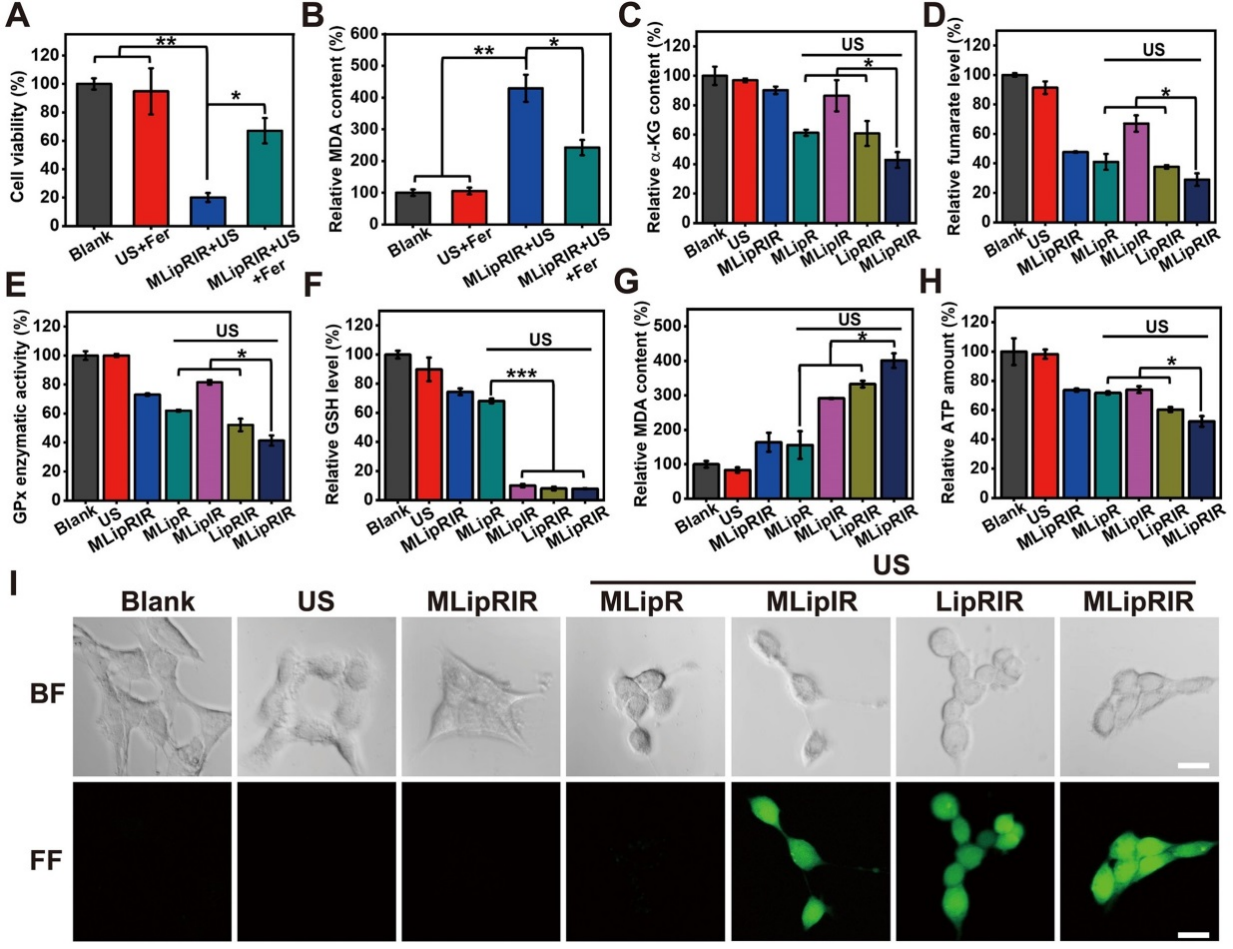
** Glutaminolysis inhibition and MLipRIR-mediated ferroptosis. (A)** Viability of 4T1 cells after treatment with MLipRIR NPs (equivalent R162 concentration: 1 µg/mL) for 4 h, followed by US irradiation (1.0 MHz, 1.5 W/cm^2^, 50% duty cycle) for 1 min. Ferrostatin-1 (Fer, a ferroptosis inhibitor, 0.5 µg/mL) was added to the applicable groups. **(B)** Relative MDA content, **(C)** relative α-kG content, **(D)** relative fumarate level, **(E)** GPx enzymatic activity, **(F)** relative GSH level, **(G)** relative MDA content and **(H)** relative ATP amount in 4T1 cells after various treatments. **(I)** Bright-field (BF) and fluorescence-field (FF) confocal images of 4T1 cells subjected to various treatments and DCFH-DA staining (scale bar: 20 µm). ^*^p<0.05, ^**^p<0.01 and ^***^p<0.001 between two groups.

Previous investigations have revealed that glutaminolysis interference by attenuating GDH1 activity may result in a decrease in GPx activity in tumor cells 10, 14. On the one hand, deactivating GPx activity would protect intracellular ROS from being consumed by reducing substances, which can indirectly augment the SDT effect. On the other hand, GPx and its isoforms are capable of catalyzing the reduction of phospholipid peroxides at the expense of GSH 57, 58. Therefore, GPx inhibition accompanied by ROS-mediated GSH consumption may lead to enhanced ferroptosis via increased lipid peroxidation. Taken together, MLipRIR-mediated GDH1 inhibition plays a key role in breaking tumor redox homeostasis. To verify our hypothesis, ferrostatin-1 (Fer), a pharmacological inhibitor of ferroptosis, was used to rescue the cells from death via this pathway. Subject to the treatment of “MLipRIR + US”, the presence of Fer significantly protected tumor cells from ferroptosis, as evidenced by the remarkably higher cell viability than that void of Fer (**Figure 3A**). Moreover, the best ferroptosis induced by “MLipRIR + US” was further verified by the highest lipid peroxidation level based on the malondialdehyde (MDA) assay (**Figure 3B**). Liperfluo is used to monitor lipid peroxidation in ferroptosis research and can specifically react with lipid hydroperoxides to form fluorescent Liperfluo-OX. A distinctly robust fluorescent response of LiperFluo was observed in the cells treated with “MLipRIR + US”, further validating the most significant ferroptosis status (**Figure S9**).

Thus far, previous findings have elucidated that both apoptosis and ferroptosis play important roles in MLipRIR-mediated tumor cell inhibition through intracellular redox dyshomeostasis. To affirm the effective inhibition of the glutaminolysis pathway, the levels of critical intermediate signaling proteins involving α-KG and fumarate were determined in cells after various treatments. The minimum α-KG level of 42.9% was unveiled from the treatment regimen of “MLipRIR + US”, benefitting from the most efficient R162 delivery into mitochondria by virtue of organelle targeting and US-triggered drug release (**Figure 3C**). As anticipated, the changing trend of fumarate, as the downstream metabolite, was similar to that of α-KG (**Figure 3D**). Importantly, the change in total GPx enzymatic activity was synchronous with the fumarate level, since fumarate can bind to and potentiate the ROS scavenging activity of GPx (**Figure 3E**). Moreover, compared with the blank control, the intracellular GSH level was dramatically decreased after administration of MLipRIR, explicitly indicating effective redox homeostasis disorder by glutaminolysis inhibition. The most notable GSH consumption (7.9% residual) was found in the “MLipRIR + US” group, which was attributed to oxidization by massive ROS levels produced by SDT (**Figure 3F**). Consistent with the intracellular GSH level, the most remarkable lipid peroxidation arising from severe redox dyshomeostasis was similarly discovered in the “MLipRIR + US” group (**Figure 3G**). Upon glutaminolysis suppression, such treatment also resulted in a tremendous decrease in intracellular ATP, owing to the obstruction of α-KG synthesis, a precursor of ATP in the TCA cycle (**Figure 3H**). A decrease in ATP production concurrently contributed to the additive tumor cell starving effect from energy deprivation 59. In addition, a 2'-7'dichlorofluorescin diacetate (DCFH-DA) ROS assay was carried out to evaluate intracellular ROS levels after various treatments. The most intense green fluorescence in the “MLipRIR + US” group indicated the maximum ROS accumulation, which was attributed to the protection of SDT-mediated production of ROS by R162-induced GDH1 inhibition (**Figure 3I and S10**).

### Release of ICD-associated DAMPs

**Figure 4:**
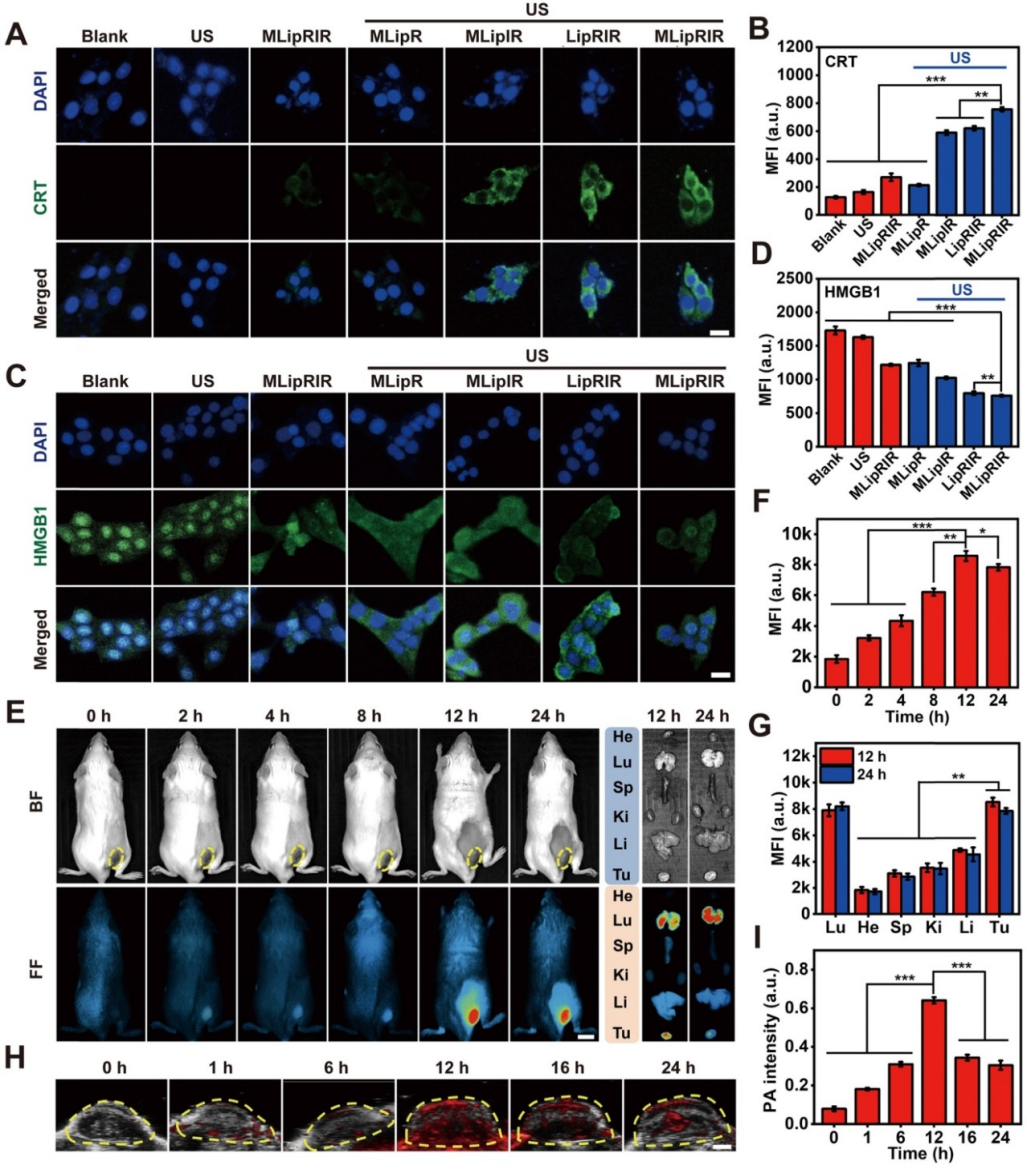
** Enhanced DAMP release *in vitro* and biodistribution of MLipRIR NPs *in vivo*. (A)** Confocal microscopic image indicating CRT exposure on 4T1 cells after various treatments (scale bar: 20 µm). **(B)** Mean fluorescence intensity (MFI) of exposed CRT. **(C)** Confocal microscopic image illustrating HMGB1 release from 4T1 cells after different treatments (scale bar: 20 µm). **(D)** MFI of remnant HMGB1 in the nuclei of 4T1 cells. **(E)** NIR fluorescence image of tumor-bearing mice at 0, 2, 4, 8, 12 and 24 h after intravenous injection of MLipRIR NPs (100 µL, 3 mg/mL) (scale bar: 1 cm), and *ex vivo* microscopy of vital organs and excised tumor (He: heart, Li: liver, Sp: spleen, Lu: lung, Ki: kidney; Tu: tumor). **(F)** MFI of the tumor region at different time points corresponding to (E). **(G)** MFI of vital organs and excised tumor harvested at 12 and 24 h corresponding to (E). **(H)** PA images of corresponding tumorous tissue at 0, 6, 16 and 24 h postinjection (scale bar: 1 cm). **(I)** PA images of PA signal harvested at 12 and 24 h corresponding to (H). ^*^p<0.05, ^**^p<0.01 and ^***^p<0.001 between the two groups.

The release of DAMPs by tumor cells succumbing to ICD contributes to the activation of the immune response by establishing a productive interface for antigen presentation. Whether MLipRIR NPs can effectively trigger ICD through the promoted release of DAMPs was thereby explored *in vitro*. Calreticulin (CRT), originally localized to the endoplasmic reticulum (ER), is responsible for instructing the conformation of glycoproteins, and translocation of CRT from the ER to the cellular membrane is deemed the hallmark of DAMP release to promote dendritic cell (DC) maturation. Specifically, surface-exposed CRT can bind to CD91 on APCs to allow the secretion of cytokines and accumulation of T helper (Th) cells 60. Hence, CRT exposure on the surface of tumor cells was observed and semiquantified by confocal imaging upon immunofluorescence staining. Significantly enhanced CRT expression was found in the cells treated with “MLipIR”, “LipRIR” and “MLipRIR” under US irradiation, as evidenced by strong green fluorescence emission (**Figure 4A**). In particular, the CRT level in the “MLipRIR + US” group increased by 5.1-fold compared with that in the blank control, arising from the intense redox dyshomeostasis mediated by combined glutaminolysis inhibition/SDT and assisted by mitochondrial targeting (**Figure 4B**). Alternatively, CRT exposure on the cell membrane was further assessed by immunofluorescence labeling and detection by flow cytometry, and the results were consistent with the confocal microscopy findings (**Figure S11**). Therapy-induced CRT translocation onto the plasma membrane can be attributed to activation of the stress sensor protein kinase R-like endoplasmic reticulum kinase (PERK) under imbalanced intracellular redox homeostasis, which leads to the phosphorylation of eukaryotic translation initiation factor (eIF2α) 61. In another aspect, the release of HMGB1 is recognized as the late ICD event and can act as a cytokine that binds to APCs for optimal antigen presentation, leading to protective immunity 62. As shown in **Figure 4C**, nuclear HMGB1 was moderately translocated and released into the cytoplasm in the “MLipRIR”, “MLipR + US” and “MLipIR +US” groups, as indicated by the weak green fluorescence in the cytosol. In contrast, a more prominent HMGB1 release was found in cells treated with “LipRIR + US” and “MLipRIR + US” (HMGB1 level decreased by 54.0% and 56.1%, respectively), as demonstrated by complete fluorescence decay in both nucleic and cytoplastic regions (**Figure 4D**). DNA fragmentation, as a marker of apoptosis, may cause the release of HMGB1 from the nucleic region through the cytoplasm to the extracellular space 63. These results verified that MLipRIR could effectively trigger ICD by chronic exposure of typical DAMPs to the immune system, which is extraordinarily favorable for immune system stimulation with the aim of tumor suppression.

### FL/PA bimodal imaging

Multimodal imaging* in vivo* not only favors a better understanding of the biodistribution kinetics of nanoagents but also provides essential information to optimize the time point to apply external stimuli. By detecting the fluorescence emission of IR780, NIR fluorescence images of tumor-bearing BALB/c mice were dynamically taken after intravenous injection with MLipRIR NPs (100 µL, 3 mg/mL). Gradual drug enrichment was observed in the tumor region for up to 12 h postadministration, as demonstrated by a progressive increase in the local fluorescence intensity (**Figure 4E**). The fluorescence intensity of the tumor region reached a maximum at 12 h and subsequently decayed at 24 h postinjection (**Figure 4F**). *Ex vivo* fluorescence imaging further confirmed a tremendously high retention of MLipRIR NPs in the tumor region, attaining a peak level at 12 h. By semiquantifying the mean fluorescence intensity (MFI), significant uptake of MLipRIR NPs was found in solid tumors, which could be ascribed to the targeting of a more active mitochondrial respiratory and the EPR effect during long-term circulation (**Figure 4G**). Comparatively strong fluorescence emission was also observed in the lung region, which was attributed to the accumulation of MLipRIR with encapsulation of IR780 granules having a specific uptake affinity to lung and breast cells. In contrast, the fluorescence intensity in the brain and heart regions were tremendously attenuated at the same time point, indicating a minimal cumulative distribution of MLipRIR NPs, a phenomenon that can be interpreted as obstruction by the respective blood-brain barrier (BBB) and monolayer of tight cardiac endothelium. Taking advantage of the strong optical absorption in the NIR region, the PA imaging capacity of MLipRIR NPs was first evaluated *in vitro*. The PA signal intensity rose with an increasing concentration up to 500 µg/mL, and the correlation coefficient (R^2^) reached as much as 0.9836, suggesting a concentration-dependent PA imaging property (**Figure S12**). Then, PA imaging performance was further assessed by developing a PA graph of solid tumors in a mouse model after intravenous injection with MLipRIR NPs (**Figure 4H**). As expected, the PA signal intensity reached a peak level at 12 h postinjection, which was attributed to the highest enrichment of MLipRIR NPs at the tumor site (**Figure 4I**). Thus, the optimum time point for US irradiation was determined to be 12 h postinjection according to observations from both fluorescence and PA imaging.

### Pharmacokinetics *in vivo*

To investigate the drug bioavailability, the *in vivo* pharmacokinetics of R162 were analyzed by quantifying its blood level after intravenous administration of either free R162 molecules or MLipRIR NPs into KM mice (equivalent R162 dosage at 0.5 mg/kg, in saline). A one-compartment exponential model was developed to simulate the drug elimination kinetics with respect to free R162 administration (**Figure S13A**). The circulating half-life of R162 was calculated to be as short as 0.32 h, implying an instantaneous drug distribution in all body parts and extremely rapid blood clearance. In sharp contrast, upon intravenous injection of MLipRIR NPs, the distribution and elimination half-lives of R162 were significantly extended to 1.37 and 4.98 h by analyzing a two-compartment exponential model (**Figure S13B**). These results demonstrated that MLipRIR NPs could effectively extend the circulating half-life of R162 in blood due to their physiological stability and controlled drug release behavior.

### Inhibition of unilateral subcutaneous tumor *in vivo*

**Figure 5:**
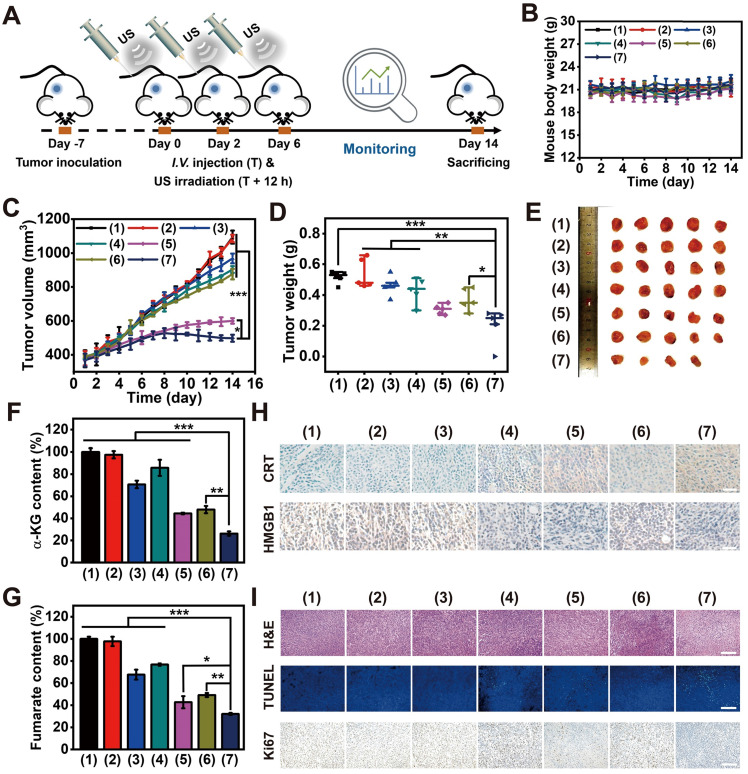
** MLipRIR mediated tumor suppression through redox dyshomeostasis *via* synergistic SDT and glutaminolysis inhibition. (A)** Schematic illustration of the treatment schedule for investigations *in vivo* (n = 5). **(B)** Time-dependent variation in mouse body weight and **(C)** changes in solid tumor volume during the treatment course of 14 days. **(D)** Tumor weight in different groups at the end of treatment. **(E)** Digital photos of a solid tumor harvested on day 14. Quantification of the expression level of **(F)** α-KG and **(G)** fumarate in tumor tissues on day 14. **(H)** Immunohistochemical staining of harvested tumor sections on day 14 to assess the expression levels of CRT and HMGB1 in the different groups (scale bar: 25 µm). **(I)** Histological analysis of tumor sections by H&E, TUNEL and Ki67 staining (scale bar: 100 µm). The groups are allocated as (1) saline, (2) US, (3) MLipR + US, (4) MLipIR + US, (5) LipRIR + US, (6) MLipRIR, and (7) MLipRIR + US. ^*^p<0.05, ^**^p<0.01 and ^***^p<0.001 between two groups.

Inspired by the admirable redox dyshomeostasis therapeutic effect *in vitro*, MLipRIR NP-mediated tumor inhibition was further validated in a 4T1 tumor-bearing mouse model. Upon the tumor reaching a volume of 250 mm^3^, mice were intravenously injected with various agents (equivalent R162 dosage at 0.5 mg/kg, in saline) on day 0. To achieve the optimum therapeutic outcome, US irradiation (1 MHz, 1.5 W/cm^2^, 50% duty cycle) was conducted at 12 h postinjection, taking into account the maximum drug enrichment in tumorous tissue. Then, R162 release was expected to be triggered by US-induced cavitation. To maximize the combinatorial treatment effect of SDT and glutaminolysis inhibition, similar drug administration followed by US irradiation was carried out on day 2 and 6 (**Figure 5A**). Thereafter, the tumor volume and mouse body weight were closely monitored over the therapeutic course of 14 days. Mouse body weight was not remarkably altered in any group, suggesting an insignificant adverse effect on animal growth (**Figure 5B**). In another aspect, tumor volume in the “MLipR + US”, “MLipIR + US” and “MLipRIR” groups synchronously increased with that in the saline group over two weeks, presenting a negligible tumor suppression effect (**Figure 5C**). In contrast, treatment with “LipRIR + US” and “MLipRIR + US” resulted in prominent tumor suppression with a tumor growth inhibition (TGI) index of 45.2% and 54.5% (on day 14), respectively. Such effective restrained tumor growth was probably due to a redox equilibrium disturbance through combinatorial SDT and glutaminolysis inhibition, and mitochondrial targeting can effectively augment this therapeutic effect. In addition, the weight of the tumors excised on day 14 followed a similar trend as the tumor volume observed in all groups (**Figure 5D-E**).

To investigate whether MLipRIR could effectively inhibit glutaminolysis, the expression of intermediate products in this metabolic pathway, including α-KG and fumarate, was quantified at the tissue level. A significant decrease in these two metabolites was found in the tumor tissue after treatment with “MLipRIR + US”, implying the most remarkable glutaminolysis inhibition through R162-mediated GDH1 deactivation (**Figure 5F-G**). Compared with the MLipRIR group, this enhanced glutaminolysis inhibition could be attributed to accelerated R162 release under repetitive US irradiation. Therefore, “MLipRIR + US” might provide the best performance in disrupting the redox balance through augmented glutaminolytic metabolism inhibition and simultaneous SDT effects. To validate the successful release of DAMPs from damaged tumor cells, CRT exposure and HMGB1 secretion in tumorous tissue were observed through immunohistochemical staining after various treatments. The most effective emission of DAMPs was observed in the “MLipRIR + US” group, which contributed to rapid inflammation activation and promoted immune‐mediated tumor elimination (**Figure 5H and S14**). Furthermore, immunofluorescence staining verified the highest quantity of antitumor CD8^+^ T cells in the cancerous tissue during the treatment with “MLipRIR + US”, which was ascribed to the promotion of DC maturation by ICD and subsequent priming of CD8^+^ T cells. Compared with the saline control, the proportion of CD4^+^ T cells was moderately increased in the same group, which might be due to the effective activation of CD4^+^ helper T cells. Concurrently, the highest expression of CD86^+^ cells indicated the highest level of primary DC maturation, which could efficiently activate T cells to induce antitumor immunity (**Figure S15**). Next, histological analysis was conducted to evaluate the pathological status of tumor tissue after various treatments (**Figure 5I**). Hematoxylin and eosin (H&E) staining suggested that the most notable tissue destruction was caused by “MLipRIR + US”, as verified by the greatest prevalence of pyknosis, karyorrhexis and karyolysis. In addition, a terminal deoxynucleotidyl transferase (TdT)-mediated dUTP nick end labeling (TUNEL) assay confirmed the most elevated apoptotic level in tumor tissue with administration of “MLipRIR + US”, as illustrated by the most pervasive green fluorescence emission in the tumor sections. Furthermore, this treatment modality also led to the most dramatic inhibition of tumor proliferation, as demonstrated by the most significant reduction in Ki67-positive cells based on Ki67 immunohistochemical staining. To evaluate long-term treatment efficacy, the survival status of tumor-bearing mice from various groups was monitored for up to 42 days, followed by calculation of the corresponding survival rates. Notably, the life span of mice subjected to MLipRIR + US treatment was obviously longer than that of mice in the other treatment groups (**Figure S16**). Altogether, these findings indicated that “MLipRIR + US” was able to effectively suppress primary tumors and simultaneously facilitate DAMP release to arouse the immune system *in vivo*.

### PD-L1 blockade synergized suppression of bilateral tumors

**Figure 6:**
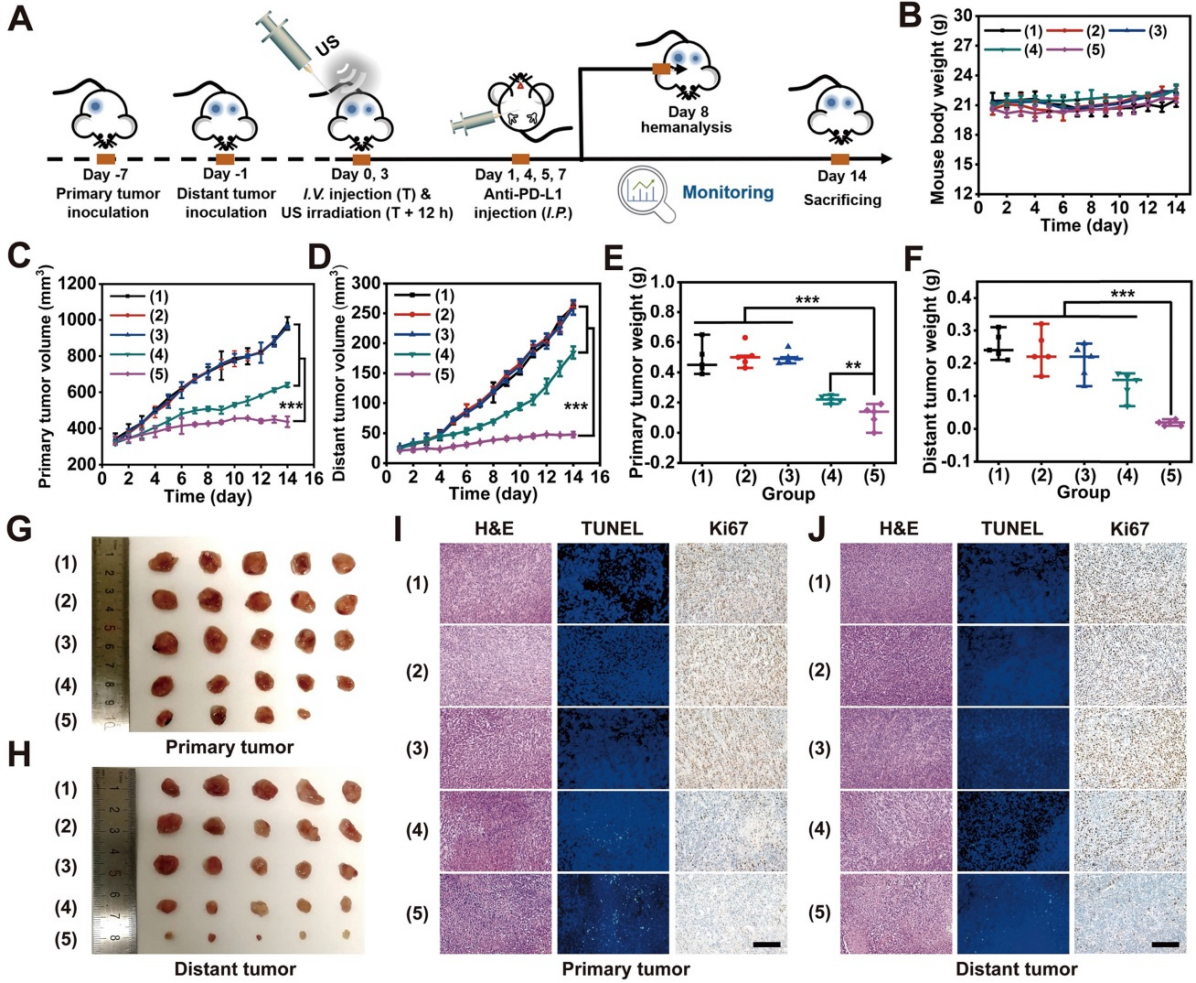
***In vivo* suppression of distant tumors through MLipRIR-mediated intracellular redox dyshomeostasis. (A)** Schematic illustration of the therapeutic procedure in animal models (n = 5). The 4T1 subcutaneous tumors were inoculated in both dorsal sides of BALB/c mice. The tumor on the right dorsal side was established as the “primary tumor” for US irradiation, and the tumor on the left dorsal side was deemed the “distant tumor” without any US treatment. **(B)** Time-dependent variation in mouse body weight over 14 days. **(C)** Primary and **(D)** distant tumor growth curve in mice subjected to various treatments. Weight of **(E)** primary and **(F)** distant tumors in different groups at the end of treatment. Digital photos of **(G)** primary and **(H)** distant tumors harvested on day 14. Histological analysis of tissue sections from **(I)** primary and **(J)** distant tumors by H&E, TUNEL and Ki67 staining (scale bar: 100 µm). The groups were assigned as (1) saline, (2) US, (3) anti-PD-L1, (4) MLipRIR NPs + US and (5) MLipRIR NPs + US + anti-PD-L1. ^**^p<0.01 and ^***^p<0.001 between two groups.

Distant metastasis always occurs during tumor development and is the primary cause of cancer death. Hence, premium cancer treatment should not only destroy the primary tumor tissue but also eradicate metastasis. PD-1/PD-L1 checkpoint blockade therapy has been verified to be a promising cancer immunotherapy strategy that exerts its effect through the enhancement of cytotoxic T-lymphocyte activity 64. In particular, during the ICD process, host antitumor immunity can be effectively activated in the TME, which transforms a noninflammatory (“cold”) tumor into a tumor enriched with a high density of infiltrating T cells (“hot”). As a result, such T cell infiltration of tumors strongly sensitizes immune checkpoint inhibition via both CTLA-4 and PD-1 blockade 65. Taking into account the effective MLipRIR-mediated ICD activation, PD-L1 blockade therapy was thereby conducted to potentiate tumor redox dyshomeostasis to simultaneously suppress both primary and distant tumors. To establish a subcutaneous tumor model, 4T1 tumor cells were first inoculated into the right dorsal side of BALB/c mice to establish a primary tumor, and a metastatic tumor mimic was similarly built in the left dorsal side after six days (**Figure 6A**). To achieve the optimum SDT effect, the primary tumor was exposed to US irradiation at 12 h after each injection of MLipRIR NPs on days 0 and 3. In light of DAMP release during redox dyshomeostasis therapy, anti-PD-L1 antibodies at a dose of 75 μg per mouse were intraperitoneally (*i.p.*) administered on day 1, 4, 5 and 7 to elicit a promoted tumor-specific immune response 46. Compared with intravenous (*i.v.*) administration of anti-PD-L1 antibody, intraperitoneal (*i.p.*) injection may decrease the systemic exposure to antibodies and further attenuate the acute systemic immune response to reduce animal lethality 66. The mice were assigned to five treatment groups: (1) saline, (2) US, (3) anti-PD-L1, (4) MLipRIR NPs + US and (5) MLipRIR NPs + US + anti-PD-L1. During the treatment period of two weeks, no obvious weight change was observed in any of the five groups, implying a minimal adverse impact on mouse growth (**Figure 6B**). In addition, US or anti-PD-L1 antibody alone exerted a negligible influence on the inhibition of both primary and distant tumors (**Figure 6C-D**). In contrast, the treatments of “MLipRIR NPs + US” and “MLipRIR NPs + US + anti-PD-L1” led to significant suppression of primary tumors, with TGIs of 34.6% and 55.6%, respectively, on day 14. An immune checkpoint inhibitor of an anti-PD-L1 antibody can optimize host immune responses following ICD induction, resulting in a much more enhanced tumor inhibition effect. Despite a certain suppression of primary tumor growth through “MLipRIR NPs + US”, the influence of this event was extremely limited toward distant tumor growth, implying that ICD triggered by redox dyshomeostasis therapy was not sufficient to provoke antitumor immunity to eliminate residual or distant tumors. Distinctly, the growth of distant tumors was almost completely inhibited upon addition of anti-PD-L1 antibody (TGI: 83.0%, on day 14). These results explicitly suggested that the immune checkpoint blockade auxiliary not only inhibited the primary tumor but also exerted abscopal and durable effects on distant tumors. The change trend observed for tumor weight agreed well with that of tumor volume, where “MLipRIR NPs + US + anti-PD-L1” led to the most significant weight reduction of both primary and distant tumors (**Figure 6E-H**). The tumor tissue was further sectioned and stained with H&E, TUNEL and Ki67 for histopathological analysis. As shown in **Figure 6I, J**, severe tissue damage was observed in both primary and distant tumors with the administration of “MLipRIR NPs + US + anti-PD-L1”, as evidenced by severe apoptosis and necrosis of tumor cells with a minimized proliferation tendency.

### Immunological response induced by redox dyshomeostasis therapy

**Figure 7:**
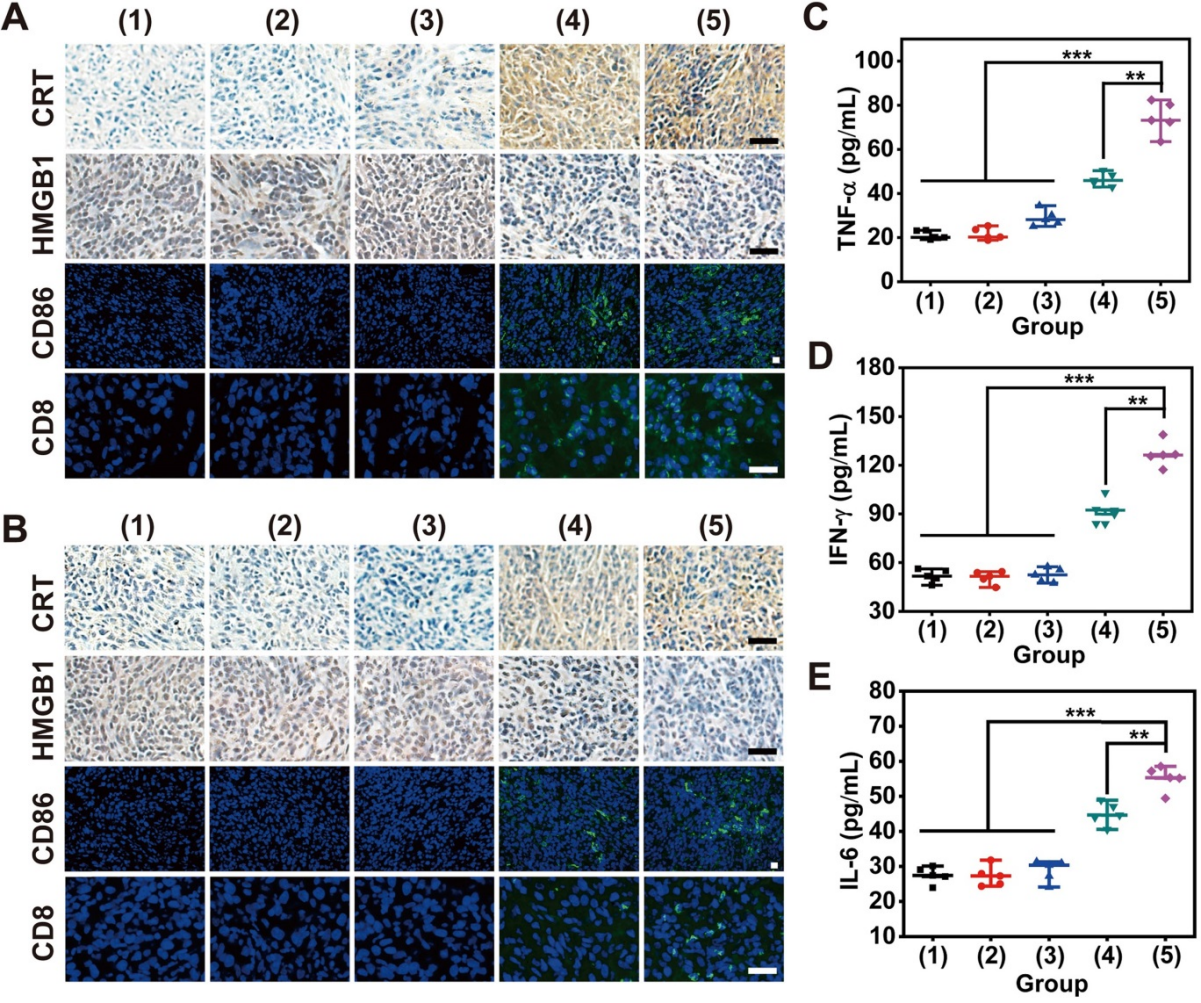
Histological analysis to indicate the expression levels of CRT, HMGB1, CD86 and CD8 in tissue sections from **(A)** primary and **(B)** distant tumors (scale bar: 25 µm). **(C)** TNF-α, **(D)** INF-γ and **(E)** IL-6 in serum on day 8 after various treatments. The groups were assigned as (1) saline, (2) US, (3) anti-PD-L1, (4) MLipRIR NPs + US and (5) MLipRIR NPs + US + anti-PD-L1.^ **^p<0.01 and ^***^p<0.001 between two groups.

To validate the emission of DAMPs from damaged tumor cells for ICD induction during bilateral tumor therapy, CRT exposure and HMGB1 release were assessed through immunohistochemical staining of tumor sections after various treatments (**Figure 7A-B**). The most significant release of DAMPs was found in both primary and distant tumors after treatment with “MLipRIR NPs + US + anti-PD-L1”, which might drive autoimmunity and promote immune‐mediated cell elimination. In addition, such treatment also resulted in the highest expression level of the DC costimulatory factor CD86, and DC maturation provided essential signals for cytotoxic T lymphocyte (CTL) activation and proliferation. Correspondingly, the largest percentage of CD8^+^ T lymphocytes, known as key effector cells in antitumor immunity, was discovered in ambilateral tumors, as revealed by the most intense green fluorescence from immunofluorescence staining. The accumulation and infiltration of CD8^+^ CTLs contributed to sweeping tumor cell elimination through the recognition of tumor-specific antigenic peptides by the T-cell receptor (TCR). The introduction of an anti-PD-L1 antibody also contributed to the most significant activation of splenic CD8^+^ CTLs and CD86^+^ DCs, a phenomenon that is highly desirable for impeding tumor metastasis and relapse (**Figure S17-S18**). Proinflammatory cytokine secretion is recognized as the typical hallmark of successful immune stimulation, and these cytokines are responsible for antigen presentation, immune effector cell activation and toxic effects. The secretion of IFN-γ from CTLs is closely associated with potent antiangiogenic activity, which is substantial to prevent tumor regression and metastasis. Consistent with the findings of DC maturation and CTL activation, “MLipRIR NPs + US + anti-PD-L1” induced the highest levels of TNF-α, IL-6 and IFN-γ in serum, which further demonstrated the enhanced immune response assisted by immune checkpoint inhibitors (**Figure 7C-E**).

### Biosafety evaluation

Reliable biosafety of MLipRIR NPs is the first requisite for their potential translation to pharmaceuticals in the future. Herein, hemocompatibility was first evaluated by exposing red blood cells (RBCs) to MLipRIR NPs (0-500 µg/mL) at 37 °C for 6 h. The hemolysis rate was calculated to be as low as 2.2% at a tremendously high drug dose of 500 µg/mL, indicating excellent hemocompatibility for long-term blood circulation (**Figure S19**). Conversely, routine blood tests were carried out after intravenous administration of MLipRIR NPs into Kunming (KM) mice, and primary indicators were all located in the normal reference range over 14 days postinjection, implying an avoidance of any disorders or disease conditions (**Figure S20**). Additionally, H&E staining of vital organs (heart, liver, lung, spleen, and kidney) displayed imperceptible pathological injuries and abnormalities in all groups, verifying the avoidance of harmful effects on normal tissues and organs (**Figure S21-S22**). Taken together, these findings elucidated the excellent biosafety of MLipRIR NPs as a promising translational medicine for antitumor applications.

The primary contribution of MLipRIR NP development can be summarized as follows. First, this study proposed a feasible strategy to effectively deliver the purpurin analog R162 into the mitochondria of tumor cells, which enabled organelle-targeted glutaminolysis inhibition to diminish GPx activity without affecting the proliferation of normal cells. The cascaded targeting effect of MLipRIR was achieved through EPR-mediated tumor enrichment and subsequent mitochondrial-specific localization guided by TPP. Second, the release of R162 from MLipRIR NPs could be accurately actuated by external US irradiation, and spatiotemporally controllable delivery of R162 was significantly beneficial for improved therapeutic efficacy, reduced side effects and enhanced patient compliance. Third, codelivery of IR780 could effectively increase intracellular oxidative stress *via* US-triggered SDT, and IR780-initiated ROS accumulation was capable of synergizing with R162-mediated AOD disruption to significantly augment redox dyshomeostasis. Moreover, IR780 encapsulation also enabled NIR fluorescence imaging-guided tumor therapy, favoring real-time monitoring of the treatment course. Finally, MLipRIR NPs displayed excellent biosafety in terms of cytocompatibility, hemocompatibility and minimal systemic toxicity due to their clinically approved liposomal formulation and lesion-targeting capability.

Thus far, liposomal products are under supervisory control by the FDA and anticipated to be approved for clinical use. In addition, R162 is a cell-permeable, nontoxic selective inhibitor of glutamate dehydrogenase 1 (GDH1), which has become increasingly attractive for inhibiting tumor growth by disrupting the anaplerotic use of glutamine in the TCA cycle 10. Despite the use of R162 being limited to fundamental research at present, its clinical translation potential has been evaluated and recognized in several recent studies 67, 68. Moreover, targeted delivery of IR780 has attracted widespread attention for clinical applications, owing to its ability to solve problems related to poor aqueous stability and adverse side effects, which may provide critical clinical benefits 69, 70. Considering all these findings, the rational design of MLipRIR may enable the potential use of such nanomedicine be for clinical tumor therapy, providing more effective therapeutic modalities for cancer patients.

## Conclusion

In summary, MLipRIR NPs with excellent mitochondrial targeting properties were synthesized to disrupt intracellular redox hemostasis through both glutaminolysis inhibition and US-activated ROS generation. High ROS levels trigger deleterious cell apoptosis by disrupting mitochondrial respiration. In addition, GPx deactivation by glutaminolysis inhibition and ROS-mediated GSH consumption led to severe ferroptosis through intense lipid peroxidation. Such synergistic apoptosis and ferroptosis caused by severe redox dyshemostasis effectively activated ICD, which could tremendously promote antitumor immunity to suppress both primary and distant tumors. Immunotherapy could be further improved by PD-L1 blockade through an elevated release of proinflammatory cytokines, APC activation and enhanced CD8^+^ CTL recruitment. FL/PA bimodal imaging mediated by MLipRIR NPs also provided useful information for drug biodistribution determination and treatment schedule formulation. Collectively, this proposed strategy based on disrupting redox homeostasis may pioneer a new avenue to treat solid tumors and metastases in future clinical translation.

## Supplementary Material

Supplementary figures.
