# Supplementary material for: Ultrasound (US)-activated redox dyshomeostasis therapy reinforced by immunogenic cell death (ICD) through a mitochondrial targeting liposomal nanosystem
Source: Theranostics. 2021 Sep 13;11(19):9470–91. doi: 10.7150/thno.62984 (PMC8490505; doi:10.7150/thno.62984)
Supplement: Supplementary file 1 — Supplementary figures. [file thnov11p9470s1.pdf]

Supporting Information

Ultrasound (US)-activated redox dyshomeostasis therapy reinforced by immunogenic cell death (ICD) through a mitochondrial targeting liposomal nanosystem

Junjie Ren[a], Jing Zhou[a], Han Liu[a], Xiaodan Jiao[a], Yang Cao[b], Zhigang Xu[a], Yuejun Kang[a,*], Peng Xue[a,*]

[a] State Key Laboratory of Silkworm Genome Biology, School of Materials and Energy, Southwest University, Chongqing 400715, China.

[b] Chongqing Key Laboratory of Ultrasound Molecular Imaging, Institute of Ultrasound Imaging, Second Affiliated Hospital, Chongqing Medical University, Chongqing 400010, China.

Corresponding author: xuepeng@swu.edu.cn (P. Xue), yjkang@swu.edu.cn (Y. Kang)

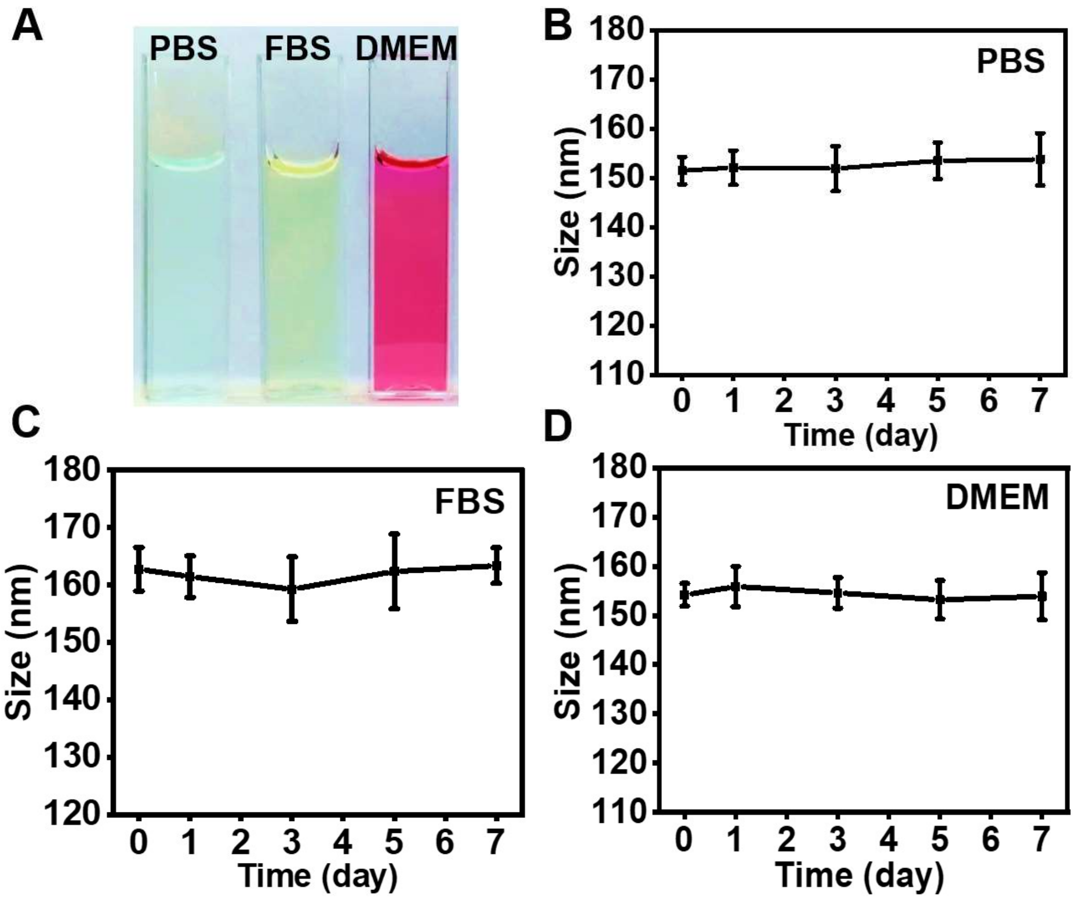

Figure S1. Long-term storage stability of MLipRIR NPs. **(A)** Digital photographs of MLipRIR NPs dispersed in PBS, FBS (10%) or DMEM (with 10% FBS). Size change of MLipRIR NPs in **(B)** PBS, **(C)** FBS (10%) and **(D)** DMEM during an incubation for seven days at 37ºC (n = 4).

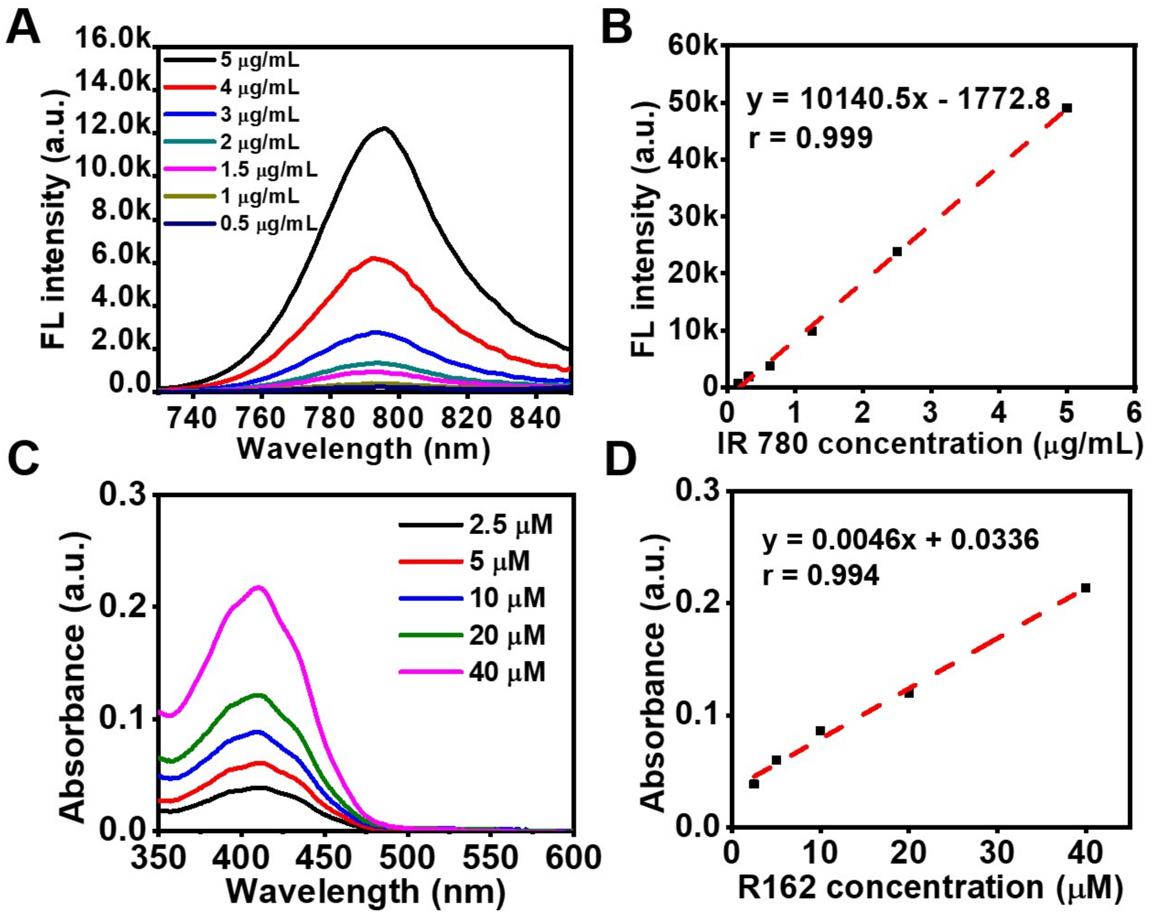

Figure S2. Calibration curves of free IR780 and R162. **(A)** Fluorescence spectra and **(B)** fluorescence intensity ($\lambda_{ex}/\lambda_{em}$ = 650/780 nm) of IR780 at various concentrations. **(C)** UV-vis-NIR absorption spectra and **(D)** absorbance (λ_{max} = 410 nm) of R162 at various concentrations.

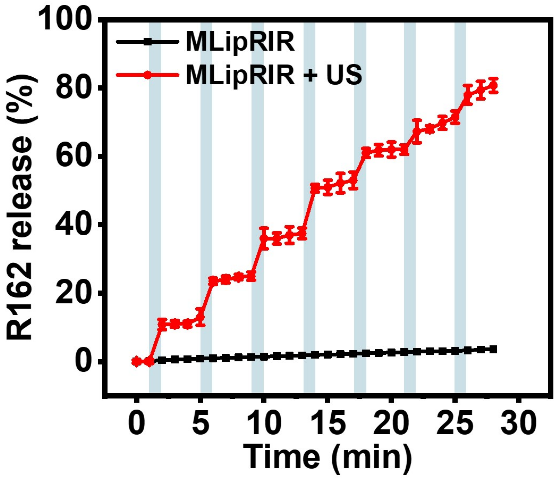

Figure S3. Cumulative release of R162 from MLipRIR NPs under the condition mimicking TME (pH: 6.8, 37 °C) *in vitro.* Blue hatched time slots represent the US-irradiation period (1.0 MHz, 1.5 W/cm^2, 50% duty cycle; 1 min irradiation for each cycle).

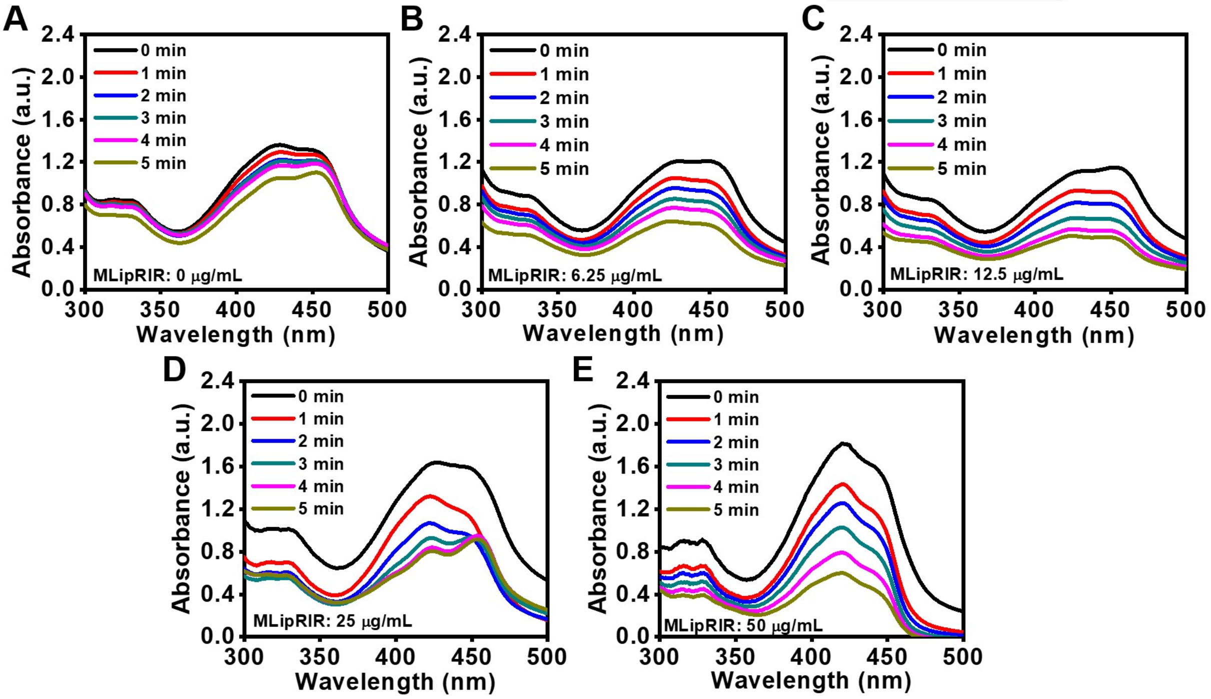

Figure S4. UV-vis absorption spectra of DPBF containing MLipRIR NPs with the concentration of **(A)** 0 μg/mL, **(B)** 6.25 μg/mL, **(C)** 12.5 μg/mL, **(D)** 25 μg/mL and **(E)** 50 μg/mL, subject to US irradiation for various periods (0-5 min) corresponding to Figure 1I.

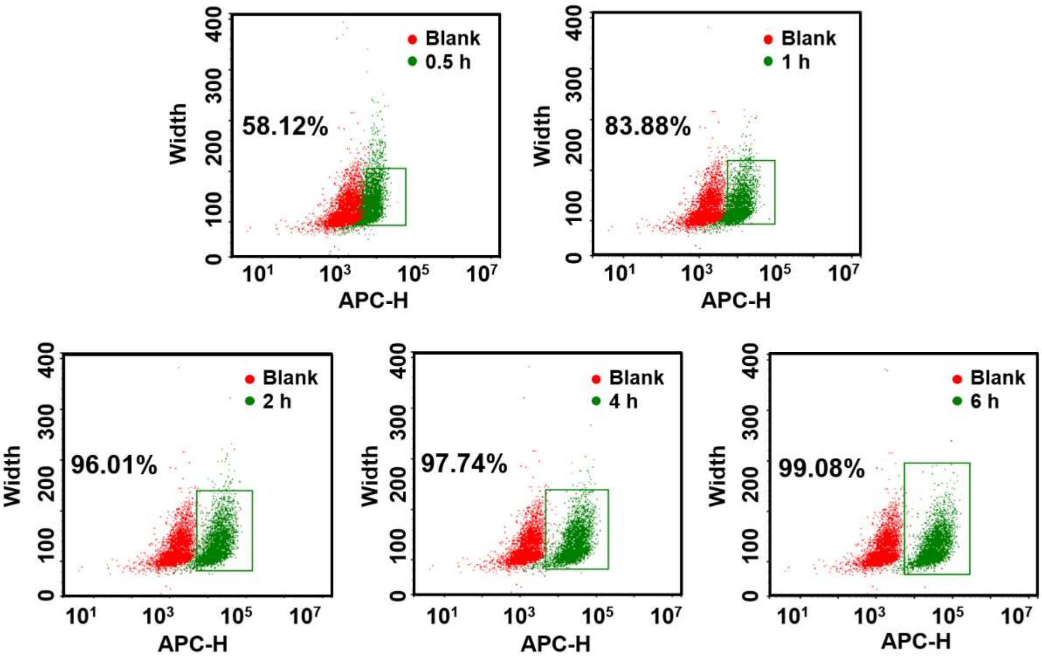

Figure S5. Flow cytometry dot plots of 4T1 cells after being incubated with MLipRIR NPs for different periods corresponding to Figure 2B.

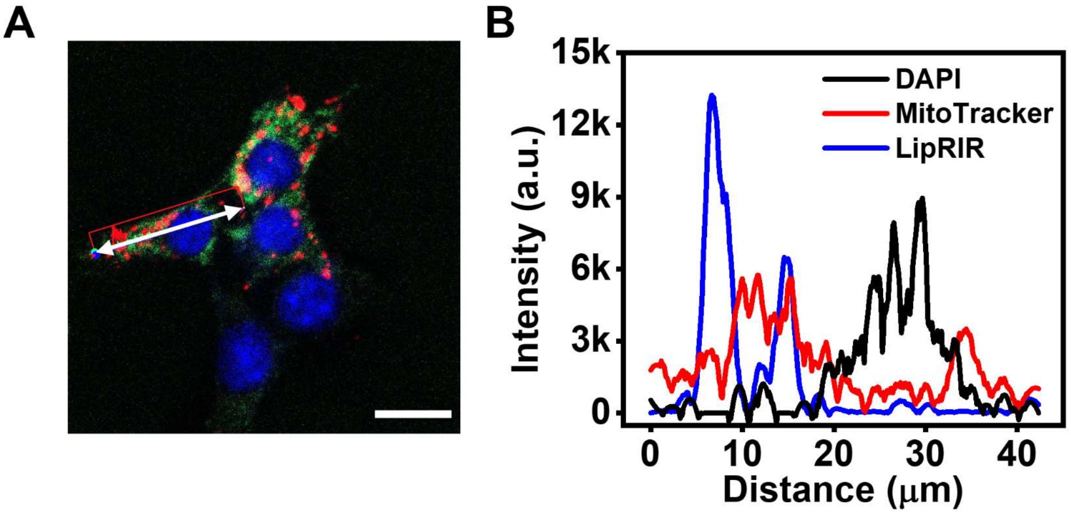

Figure S6. (A) Feature confocal image of 4T1 cells after incubation with LipRIR NPs for 4 h (scale bar: 20 μm). Fluorescence of DAPI, MitoTracker Green and LipRIR NPs was displayed by pseudo-colored blue, green and red, respectively. **(B)** Fluorescence intensity of individual DAPI, MitoTracker Green and MLipRIR NP channels along with the white auxiliary line marked in (A).

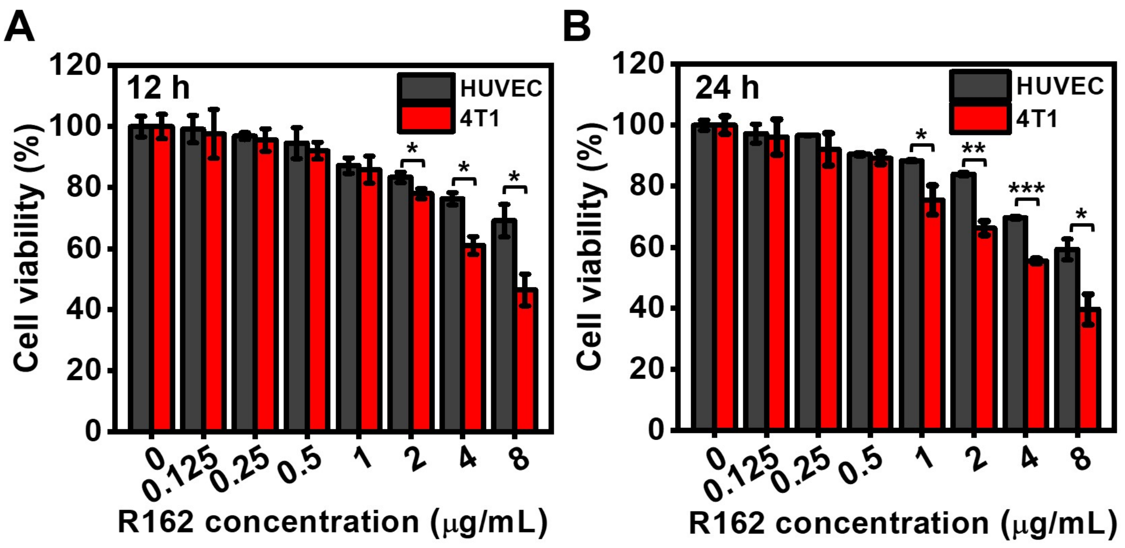

Figure S7. Viability of HUVEC and 4T1 cells after administered with MLipRIR NPs at various concentrations for **(A)** 12 h and **(B)** 24 h ($^{*}p<0.05$, $^{**}p<0.01$ and $^{***}p<0.001$ between two groups).

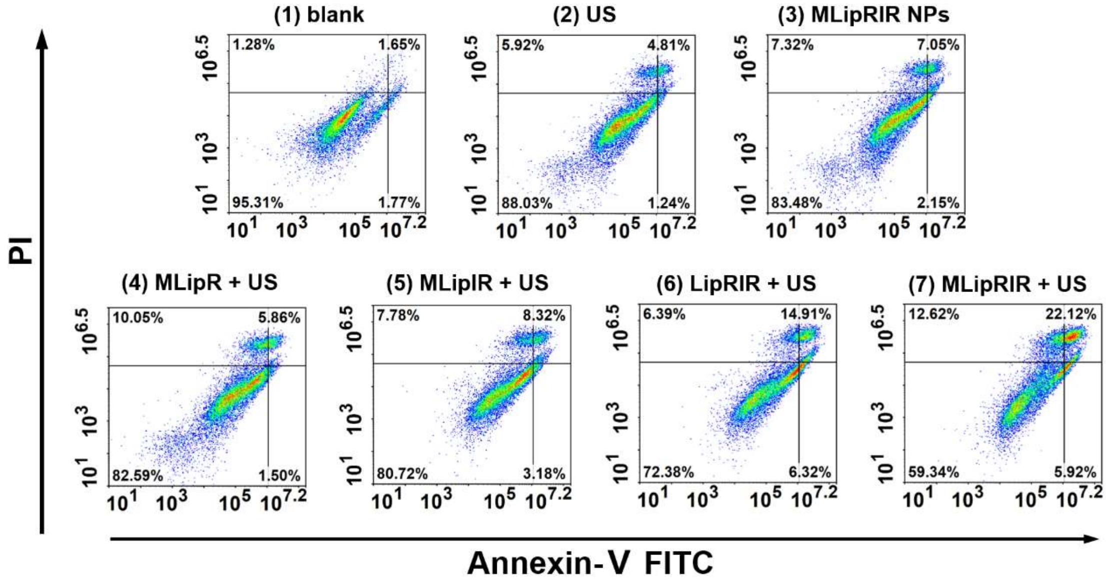

Figure S8. Apoptotic status of 4T1 cells after receiving various treatments and stained by Annexin V-FITC/PI, followed by quantitative analysis using flow cytometry.

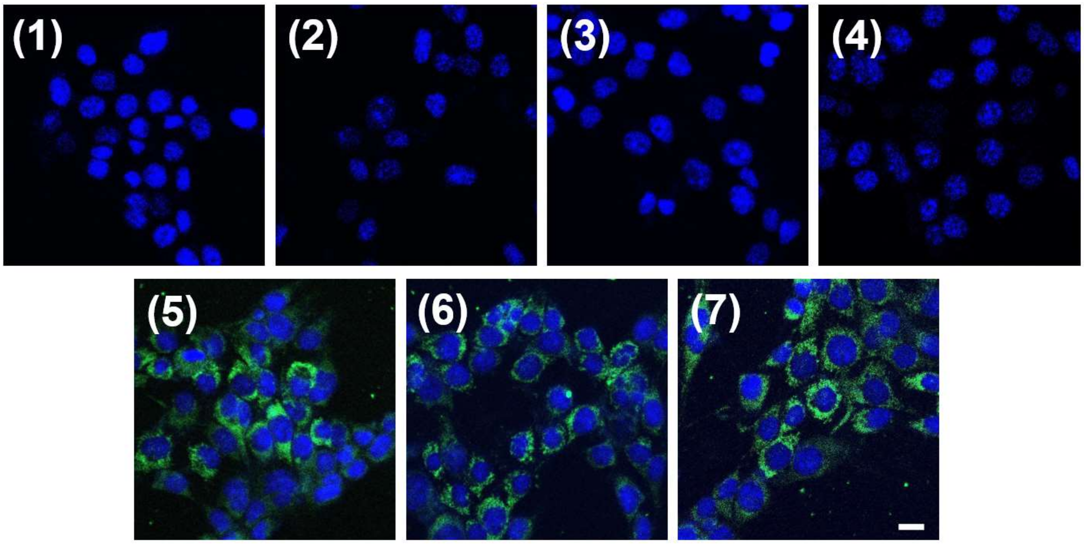

Figure S9. Confocal microscopic images of 4T1 cells pre-stained by LiperFluo and subject to different treatments corresponding to Figure 5G (scale bar: 20 μm). Groups are assigned to be (1) blank, (2) US, (3) MLipRIR NPs, (4) MLipR + US, (5) MLipIR + US, (6) LipRIR + US, and (7) MLipRIR + US.

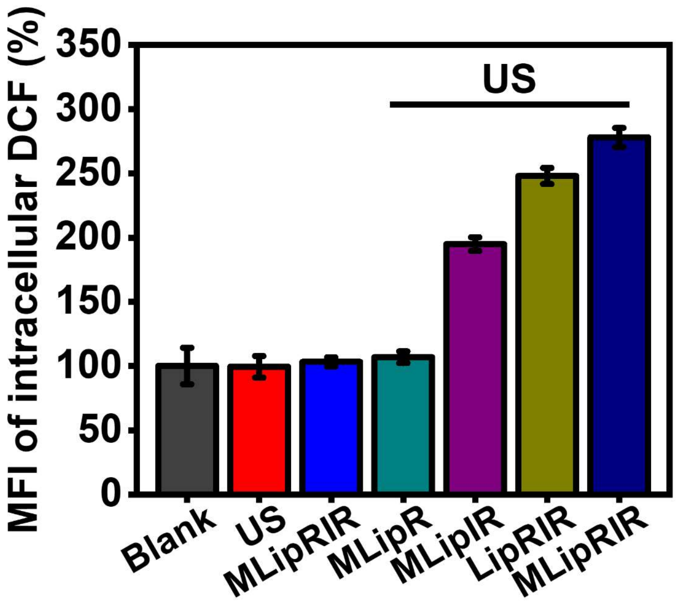

Figure S10. Mean fluorescence intensity (MFI) of intracellular DCF after various treatments toward 4T1 cells corresponding to Figure 3I.

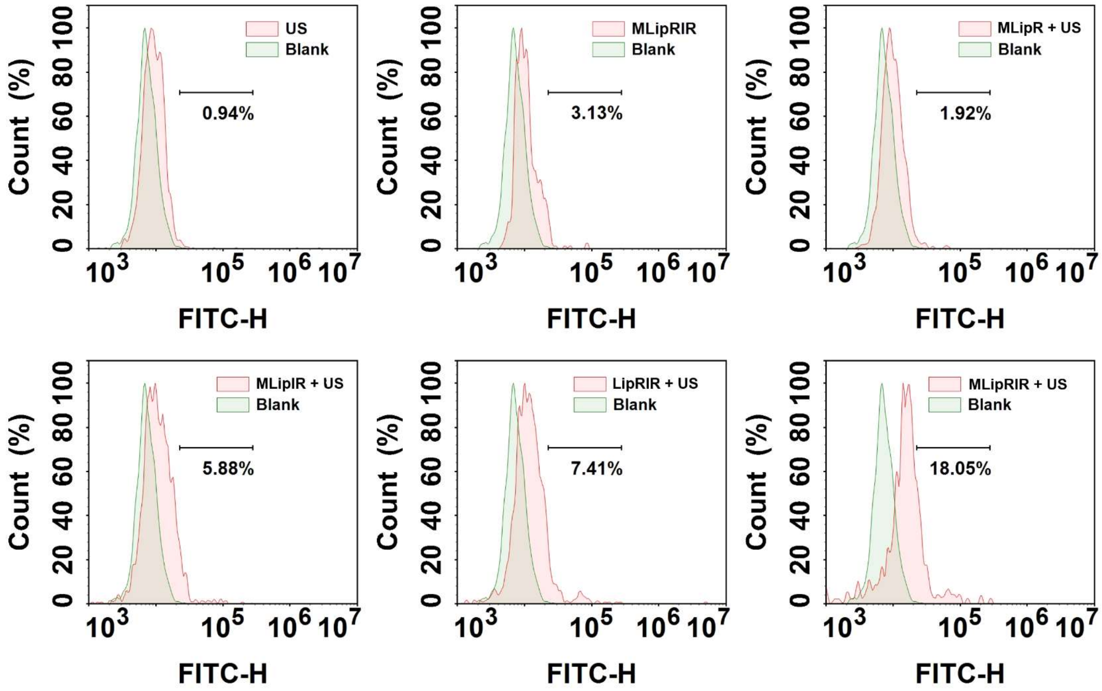

Figure S11. CRT expression level in the plasma membrane of 4T1 cells after various treatments through flow cytometry analysis.

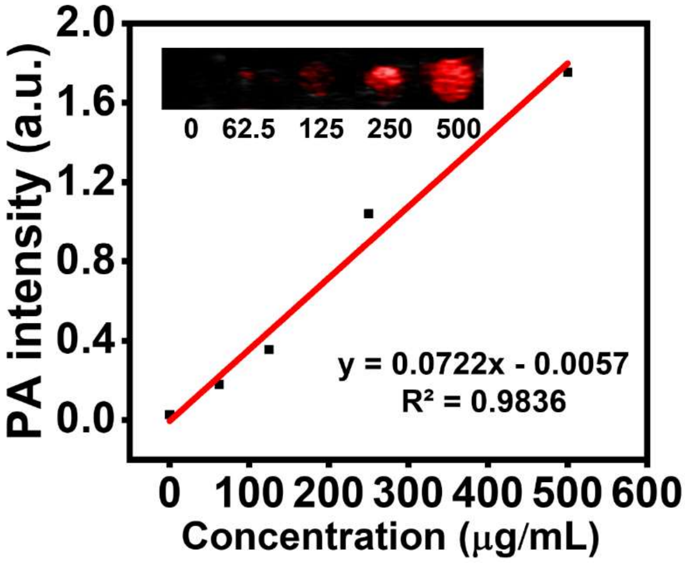

Figure S12. Calibration curve of PA intensity as a function of MLipRIR concentration (ranging up to 500 μg/mL). Inset: PA image of MLipRIR dispersion with different concentrations.

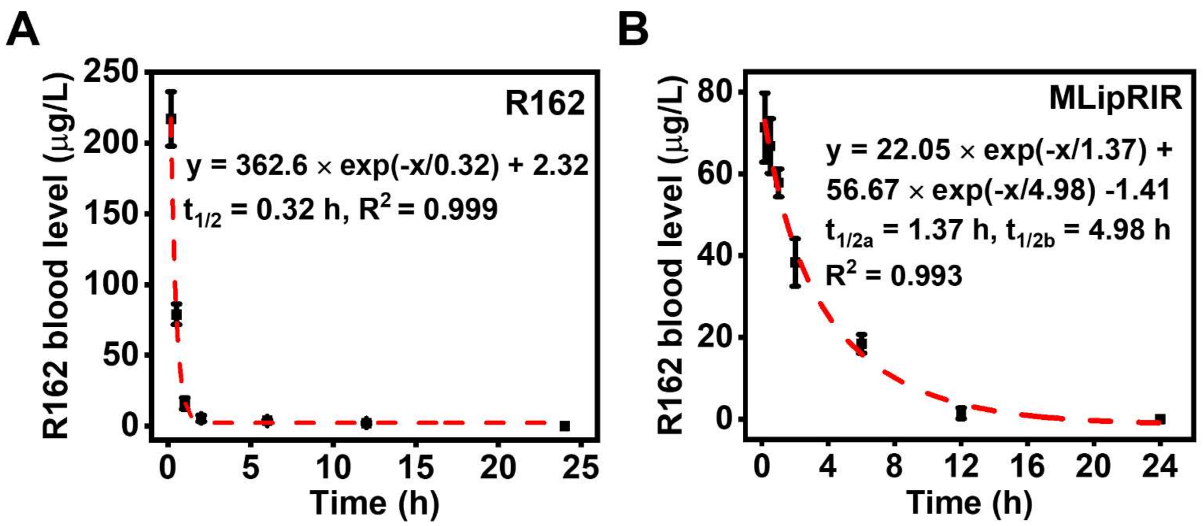

Figure S13. Pharmacokinetics of R162 *in vivo*. R162 blood level over 24 h after intravenous injection of **(A)** free R162 or **(B)** MLipRIR NPs (100 μL, equivalent R162 dosage at 0.5 mg/kg, in saline) into KM mice.

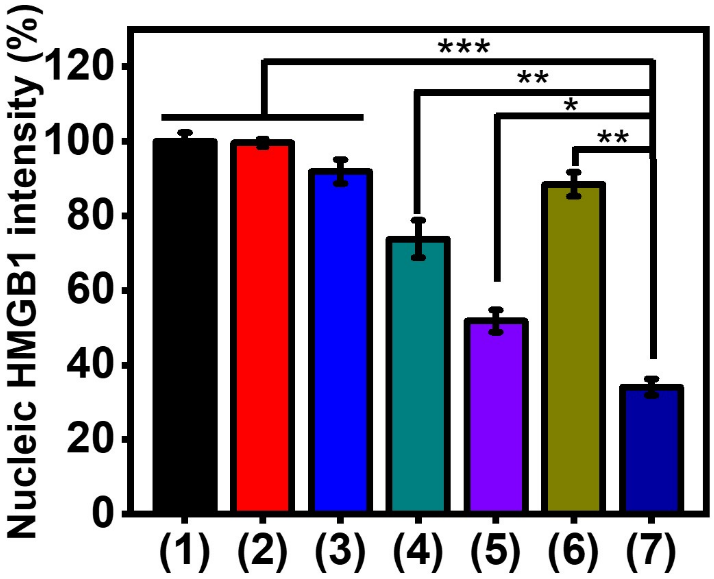

Figure S14. Quantitative remnant HMGB1 level in nucleic region of 4T1 tumor section from various groups corresponding to Figure 5H. $^{*}p<0.05$, $^{**}p<0.01$ and $^{***}p<0.001$ between two groups.

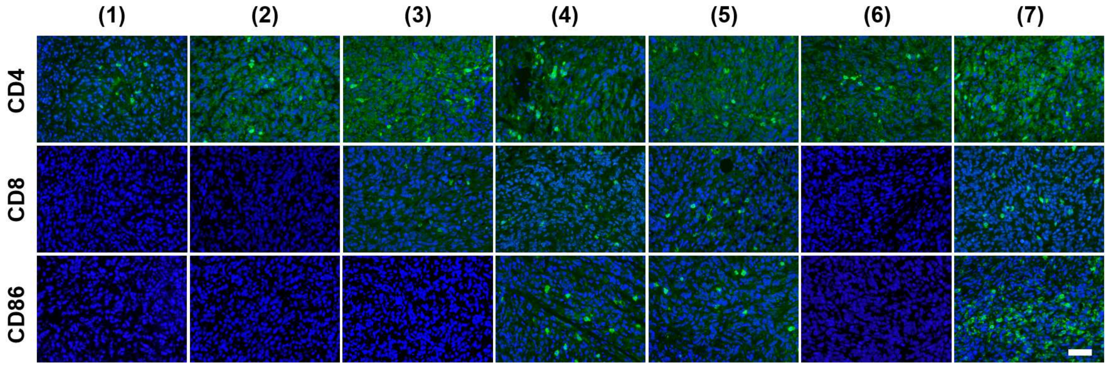

Figure S15. Representative confocal microscopic images of tumor slices with immunofluorescence staining against CD4, CD8 and CD86 on day 14 after various treatments, corresponding to the groups in Figure 5 (scale bar: 50 μm).

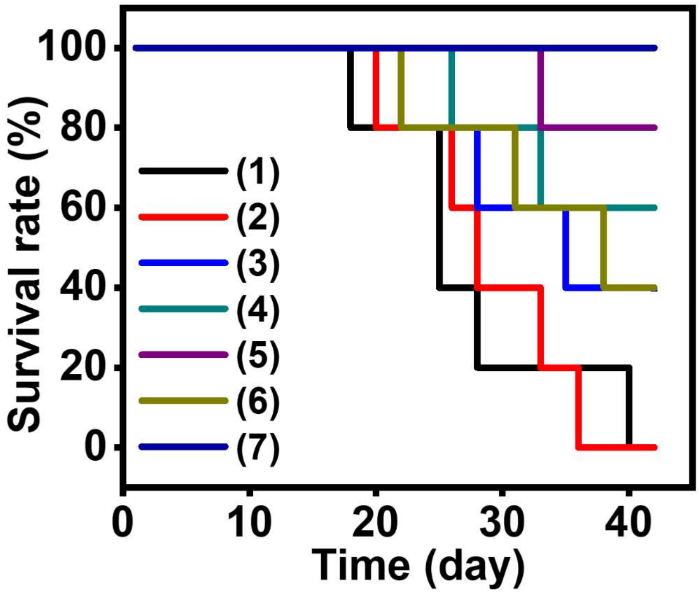

Figure S16. Survival rates of 4T1 tumor-bearing mice subject to various treatments corresponding to Figure 5 (n = 5).

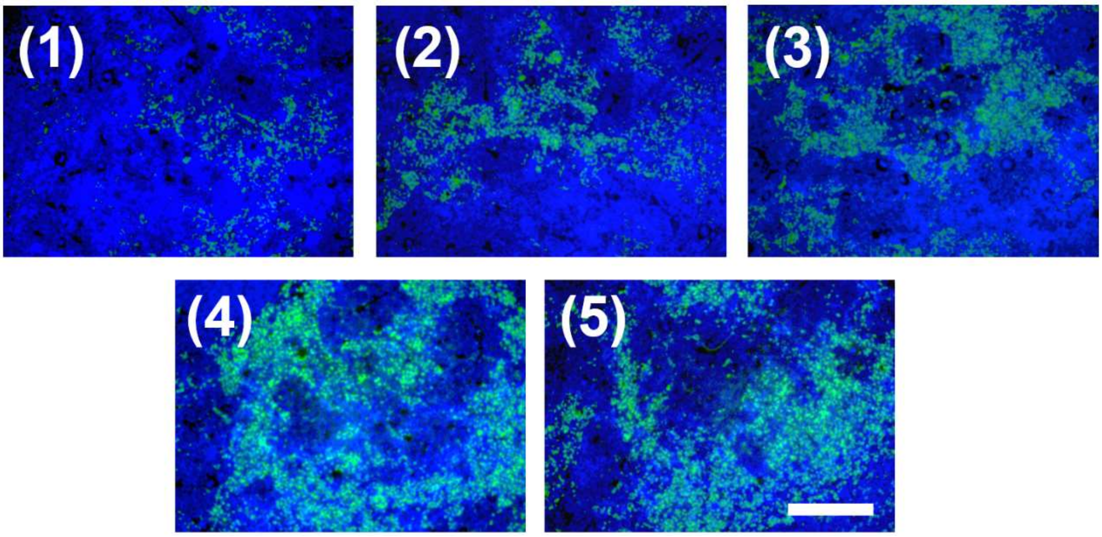

Figure S17. Representative confocal images of spleen slices with immunofluorescence staining against CD8 on day 14 after various treatments, corresponding to the groups in Figure 6 (scale bar: 100 μm).

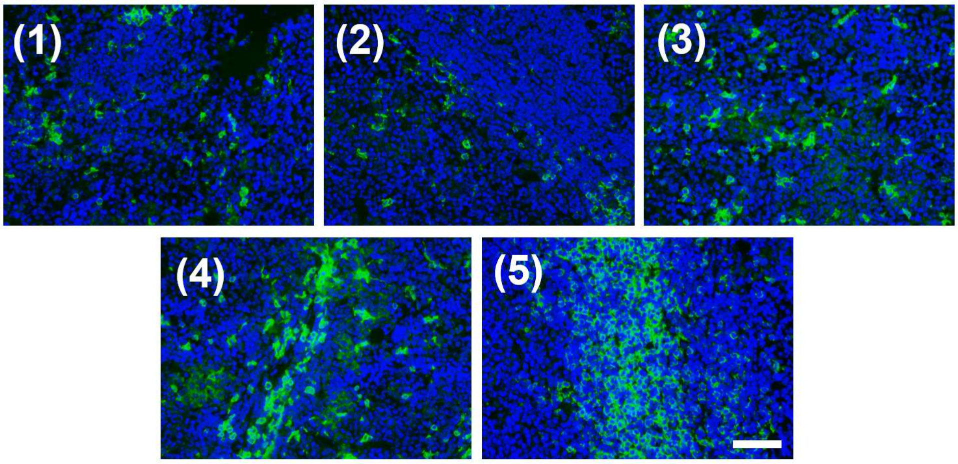

Figure S18. Representative confocal microscopic images of spleen slices with immunofluorescence staining against CD86 on day 14 after various treatments, corresponding to the groups in Figure 6 (scale bar: 50 μm).

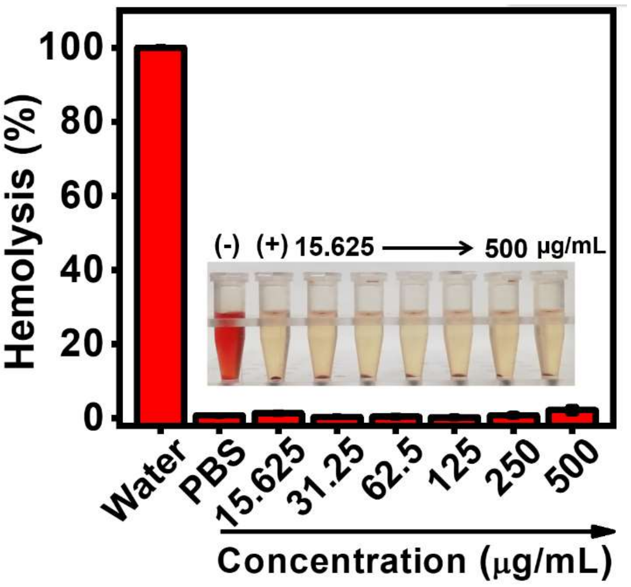

Figure S19. Hemolytic rate of red blood cells (RBCs) after incubating with MLipRIR NPs at different concentrations for 6 h at 37 ºC. DI water and PBS served as positive and negative references, respectively. Inset: photograph of the centrifuge tubes containing various samples.

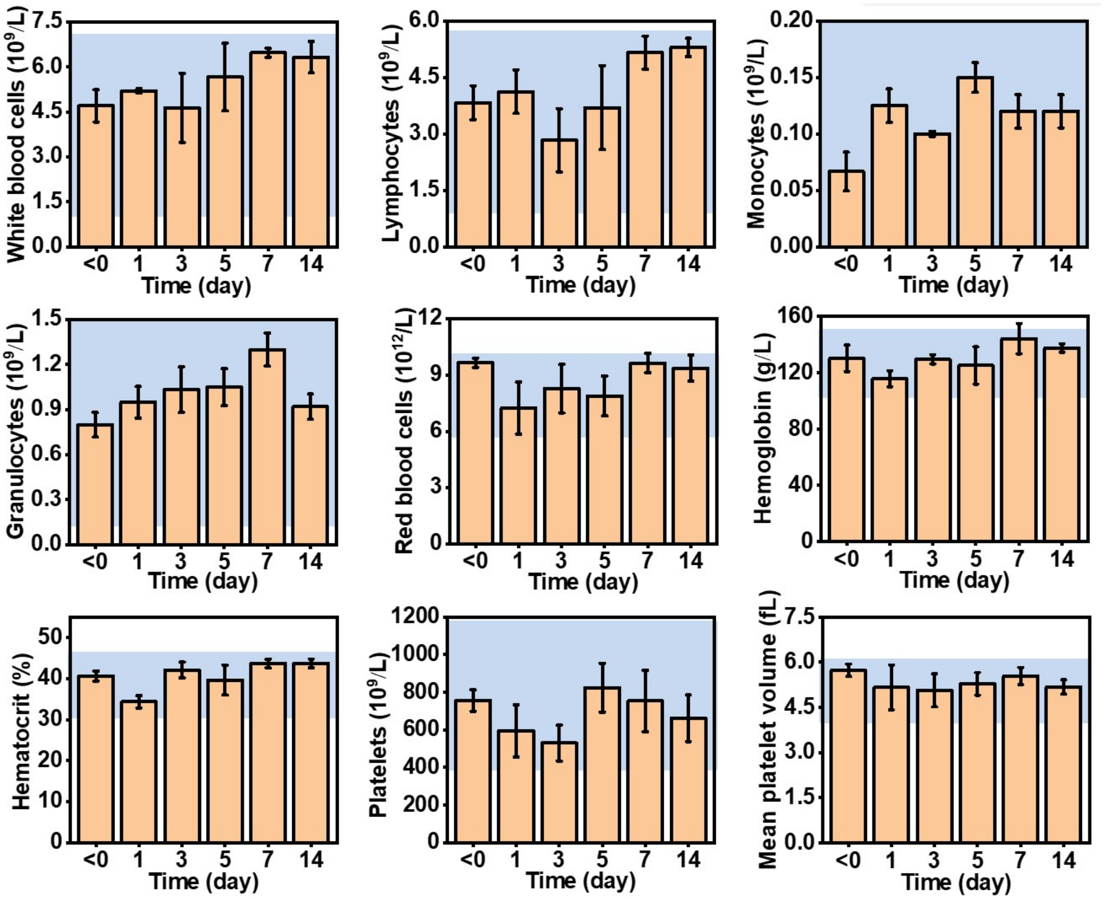

Figure S20. Primary indicators of routine blood test after KM mice being injected with MLipRIR NPs (equivalent R162 concentration at 0.5 mg/kg). The blue hatched areas represent the normal reference ranges of hematology data of healthy mice.

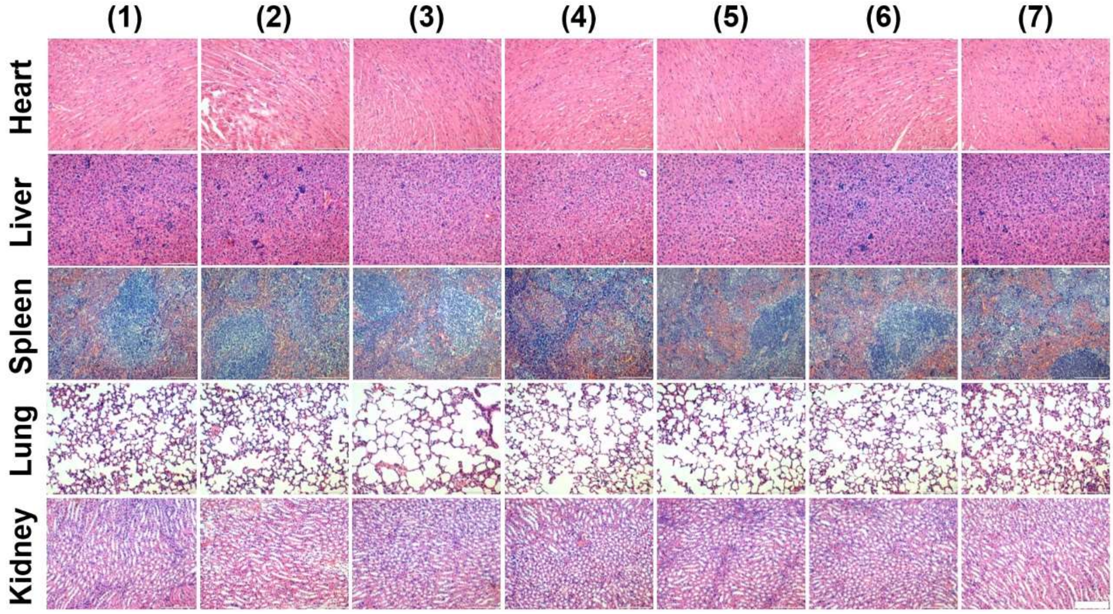

Figure S21. Histopathological analysis of vital organs (heart, liver, spleen, lung and kidney) harvested on day 14 through H&E staining, corresponding to the groups in Figure 5 (scale bar: 100 μm).

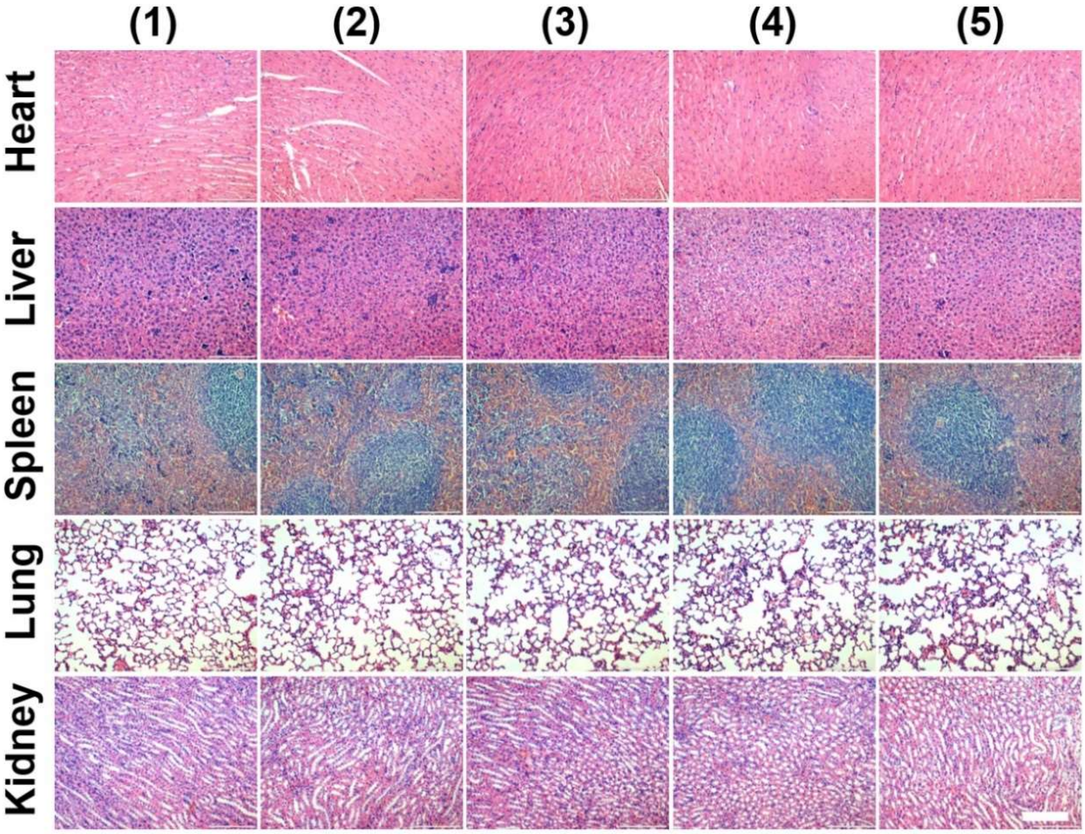

Figure S22. Histopathological analysis of vital organs (heart, liver, spleen, lung and kidney) harvested on day 14 through H&E staining, corresponding to the groups in Figure 6 (scale bar: 100 μm).
